# Cellular Size as a Means of Tracking mTOR Activity and Cell Fate of CD4+ T Cells upon Antigen Recognition

**DOI:** 10.1371/journal.pone.0121710

**Published:** 2015-04-07

**Authors:** Kristen N. Pollizzi, Adam T. Waickman, Chirag H. Patel, Im Hong Sun, Jonathan D. Powell

**Affiliations:** Department of Oncology, Sidney Kimmel Comprehensive Cancer Center, Johns Hopkins University School of Medicine, Baltimore, Maryland, United States of America; Purdue University, UNITED STATES

## Abstract

mTOR is a central integrator of metabolic and immunological stimuli, dictating immune cell activation, proliferation and differentiation. In this study, we demonstrate that within a clonal population of activated T cells, there exist both mTOR^hi^ and mTOR^lo^ cells exhibiting highly divergent metabolic and immunologic functions. By taking advantage of the role of mTOR activation in controlling cellular size, we demonstrate that upon antigen recognition, mTOR^hi^ CD4+ T cells are destined to become highly glycolytic effector cells. Conversely, mTOR^lo^ T cells preferentially develop into long-lived cells that express high levels of Bcl-2, CD25, and CD62L. Furthermore, mTOR^lo^ T cells have a greater propensity to differentiate into suppressive Foxp3+ T regulatory cells, and this paradigm was also observed in human CD4+ T cells. Overall, these studies provide the opportunity to track the development of effector and memory T cells from naïve precursors, as well as facilitate the interrogation of immunologic and metabolic programs that inform these fates.

## Introduction

The evolutionarily conserved serine/threonine kinase mammalian Target of Rapamycin (mTOR) is a central integrator of environment cues and intracellular stress signals, dictating the course of cellular growth, proliferation and differentiation [1]. The mTOR kinase forms two distinct signaling complexes, mTORC1 and mTORC2, with distinct upstream activators and discrete downstream targets [2]. mTORC1, characterized by the scaffolding proteins Raptor and PRAS40 along with mLST8 and Deptor, is canonically activated upon stimulation of PI3-kinase [3]. This occurs in part through the phosphorylation and inactivation of the mTORC1-inhibitory proteins TSC1/2 [2,4,5]. These events result in the phosphorylation of the canonical mTORC1 substrates S6 kinase and 4EBP1, causing enhanced translation of mRNA transcripts with TOP or TOP-like motifs, increased mitochondrial biogenesis, and enhanced expression of the critical metabolic transcription factors Myc and HIF-1α [6–9]. mTORC1 activity plays a critical role in regulating cell size, particularly in promoting the increase in cell size upon activation [10].

In T cells, the kinase activity of mTOR has been shown to be modulated by numerous immunological stimuli including TCR ligation, co-stimulation/co-inhibition, cytokine/chemokine exposure, and adhesion molecule engagement [11]. Indeed, mTOR has emerged as an important integrator of cues from the immune microenvironment to guide T cell effector and memory differentiation [12,13]. In the case of CD4+ T cells, pharmacological inhibition of mTOR signaling or genetic deletion of the mTOR gene results in a severe defect in the ability of a naïve CD4+ T cell to adopt an effector phenotype following activation [14–18]. Instead, CD4+ T cells activated in the absence of mTOR signaling adopt a default Foxp3+ regulatory cell phenotype [14]. In contrast, studies utilizing CD4+ T cells lacking components of either the mTORC1 or mTORC2 complex have revealed that mTORC1 signaling is required for the development of IFN-gamma producing Th1 cells and IL-17 producing Th17 cells, while mTORC2 is required for the development of IL-4 producing Th2 cells [19,20]. In addition to regulating CD4+ T cell effector differentiation, mTOR has been shown to play a central role in regulating the development of long-lived memory CD8+ T cells [21–23].

The data derived from knockout mice and pharmacologic inhibitors suggests that the differential activation of mTOR signaling during an immune response plays an important role in dictating fate decisions for activated T cells. To this end, we sought to identify cells based on their level of mTOR activity and then track their fate. In this study, we demonstrate that cell size can be used as a surrogate indicator of mTOR activity in recently activated T cells, allowing for the isolation of mTOR^hi^ and mTOR^lo^ populations with divergent differentiation, metabolic and survival potential. CD4+ mTOR^hi^ T cells preferentially develop into short-lived, terminally differentiated effector cells with a robust metabolic phenotype and large proliferative capacity. Alternatively, mTOR^lo^ CD4+ T cells are less proliferative, less glycolytic, and demonstrate a long lived phenotype. We also find that enriched within this population of mTOR^lo^ cells, are suppressive Foxp3+ regulatory T cells, and this finding was recapitulated in human CD4+ T cells.

## Materials and Methods

### Animals

Mice were kept in accordance with guidelines of the Johns Hopkins University Institutional Animal Care and Use Committee. 5c.c7 Rag^–/—^and OT-II mice were purchased from Taconic. C57BL/6 mice, CD90.1 mice, CD4 Cre recombinase mice, Foxp3^GFP+^ mice, and mice with *lox*P-flanked *Raptor* alleles were obtained from Jackson Laboratories. Mice with *lox*P-flanked *Tsc2* alleles were generated by the laboratory of M. Gambello [24]. 5c.c7 Rag^–/—^mice were derived from a B10.A background, while all other WT and OT II+ mice described in this manuscript originate from the C57BL/6 strain.

### Media and cell culture

Primary cell culture was performed in complete media consisting of 45% RPMI + 45% Clicks media supplemented with 10% FCS, L-glutamine, ßME and antibiotics. Metabolic flux analysis was performed in complete Seahorse media (Seahorse Bioscience, 102365) supplemented with 25mM D-Glucose, 1mM Na-Pyruvate and 2mM L-glutamine. 5c.c7 Rag^–/—^splenocytes were stimulated with 0.5ug/ml Pigeon CytochromeC (PCC) peptide (JHU CORE synthesis). C57BL/6 WT splenocytes were stimulated with 0.1–1ug/ml anti-CD3 depending on the experiment. Splenocytes derived from transgenic OT-II+ mice were stimulated with 5ug/ml OVA^323–339^ peptide (Anaspec).

### Proliferation assay

Sorted cells were cultured for 24hrs in complete media supplemented with 1ng/ml IL-2, then equivalent cell numbers were loaded in a 96 well plate and treated with 1μCi ^3^H-thymidine for 18hrs. ^3^H incorporation was measured using a PerkinElmer 1450 Microbeta counter.

### Cell cycle analysis

For the determination of intracellular DNA content, cells were washed with PBS and fixed for 30min at 4°C in ice-cold 70% EtOH. Cells were then washed with PBS, and treated with 50U of RNase 1F (New England Biolabs M0243) for 10minutes at room temperature, and suspended in a 50ug/ml solution of propidium iodide. Cell cycle progression was determined by flow cytometry.

### Flow cytometry and cell sorting

All flow cytometry experiments were performed on a BD FACS Calibur or LSR II, and analyzed using FlowJo software. Cell sorting was performed on a BD FACS Aria II.

The following antibodies for flow cytometry of murine samples were purchased from BD Biosciences: anti-CD25 (PC61), anti-CD4 (RM4-5), anti-CD69 (HI.2F3), anti-CD90.1 (OX-7), anti-CTLA-4 (UC10-4F10-11), and anti-CD71 (C2). The following antibodies for flow cytometry were purchased from eBioscience: anti-Foxp3 (FJK-16s), anti-CD11a (M17l4), anti-CD62L (MEL-14), anti-CD39 (24DMS1), and anti-GITR (DTA-1). Anti-ribosomal S6 Ser235/236 (2211), anti-ribosomal S6 Ser240/244 (5364), and anti-p70 S6K T389 (9205) were purchased from Cell Signaling Technologies. Anti-Bcl-2 (BCL/10C4) and anti-CXCR3 (CXCR3-173) were purchased from Biolegend. As for antibodies against human: anti-HLA-DR, anti-CD38 (HB7), and anti-Foxp3 (259D/C7) were purchased from BD Biosciences. Anti-CD4 (OKT4), anti-CD127 (A019D5) and anti-CD25 (BC96) were purchased from Biolegend. Intracellular Foxp3 and Bcl-2 staining was performed using the Fixation/Permeabilization kit (eBioscience). For assessment of intracellular phospho-proteins, cells were permeabilized with methanol and fixed with 2% formalin prior to staining. However, for assessment of mTORC1 activity in the activated human samples and in activated cells cultured in rapamycin or TGF-ß, phosphorylated S6 expression was assessed after fixation/permeabilzation in the eBioscience kit. Determination of mitochondrial content was determined by Mitotracker Green staining according to the manufacturer’s instructions (Invitrogen). Cell proliferation analysis was performed using CellTrace CFSE (Invitrogen) or eFluor670 proliferation dye (eBioscience) according to the manufacture’s instructions.

### Real Time PCR

RNA from stimulated cells was harvested using Trizol and cDNA generated using M-Mulv reverse transcriptase (NEB). Gene expression analysis was performed using ABI TaqMan 2X Universal Master Mix II (4440040) and rtPCR probes for IFNg (Mm99999071_m1), IL-17a (Mm00439619_m1), IL-17f (Mm00521423_m1), Bcl-2 (Mm00477631_m1), Foxp3 (Mm00475162), and IL2ra (Mm01340213_m1). ΔΔC_t_ values were normalized to levels of house keeping gene 18S ribosomal RNA (Life technologies). Analysis was performed on an ABI OneStepPlus 96 well instrument.

### Extracellular flux analysis

Cellular metabolic parameters were measured using a Seahorse Bioscience XF96 Extracellular Flux Analyzer. Activated T cells were adhered to Poly-D-lysine coated 96 well plates and briefly cultured in complete Seahorse media. Cellular metabolic parameters were assayed by sequential addition of Oligomycin (final concentration 1uM) and FCCP (final concentration 1.5uM).

### Immunoblot analysis

Immunoblot analysis was performed as previously described [25]. Antibodies used: anti-Bcl-2 (3498) (Cell Signaling Technologies), and anti-actin (8456) (Sigma).

### Suppression Assays

Splenocytes from a WT C57BL/6 mouse were stimulated for 16 hours with 0.1ug/ml anti-CD3 then sorted into mTOR^hi^ and mTOR^lo^ populations and cultured with 1ng/ml IL-2 for 72 hrs. Cells from the mTOR^hi^ and mTOR^lo^ cultures were mixed at a 1:2 ratio with naïve WT CD90.1+ CD4+ T cells stained with CFSE and stimulated with 1ug/ml anti-CD3 and irradiated APCs. The proliferation of the naïve CD4+ CD90.1+ T cells was measured by CFSE dilution by flow cytometry. For the transwell assays, mTOR^hi^ or mTOR^lo^ cells were segregated from the CFSE labeled naïve WT CD90.1+ CD4+ T cells by a Transwell permeable support 0.4uM polycarbonate membrane (Corning). Both the segregated suppressor and responder populations were co-cultured with 1ug/ml anti-CD3 and irradiated APCs.

### Human CD4+ T cell experiments

Human samples were obtained through an institutional review board approved protocol. Blood was drawn from a total of 8 healthy volunteers in 3 independent experiments. PBMCs were extracted after Ficoll separation during centrifugation (Ficoll-Paque PLUS, GE Healthcare). CD4+ T cells were purified by magnetic bead separation according to the manufacture’s instructions (Human CD4+ T cell enrichment kit, Stem Cell Technologies). Purified CD4+ T cells were stimulated 20 hrs with dynabeads human T- activator anti-CD3/anti-CD28 beads (Gibco). The 15% biggest ‘mTOR^hi^’ and smallest ‘mTOR^lo^’ CD38+, CD4+ T cells were sorted on a BD FACS Aria II. Sorted cells were cultured in media supplemented with 3,000units/ml human IL-2 (provided by the NCI) for 72 hrs. Phenotype of sorted cells was determined by flow cytometry.

### Statistical Analysis

Data are presented as means and standard deviation. Statistical results were generated using Graphpad Prism. Comparisons were calculated using unpaired T tests, and results with a p-value < 0.05 were considered statistically significant: *p<0.05, **p<0.01, ***p<0.001.

## Results

### Cell size indicates mTOR activity in recently activated CD4+ T cells

**Fig 1:**
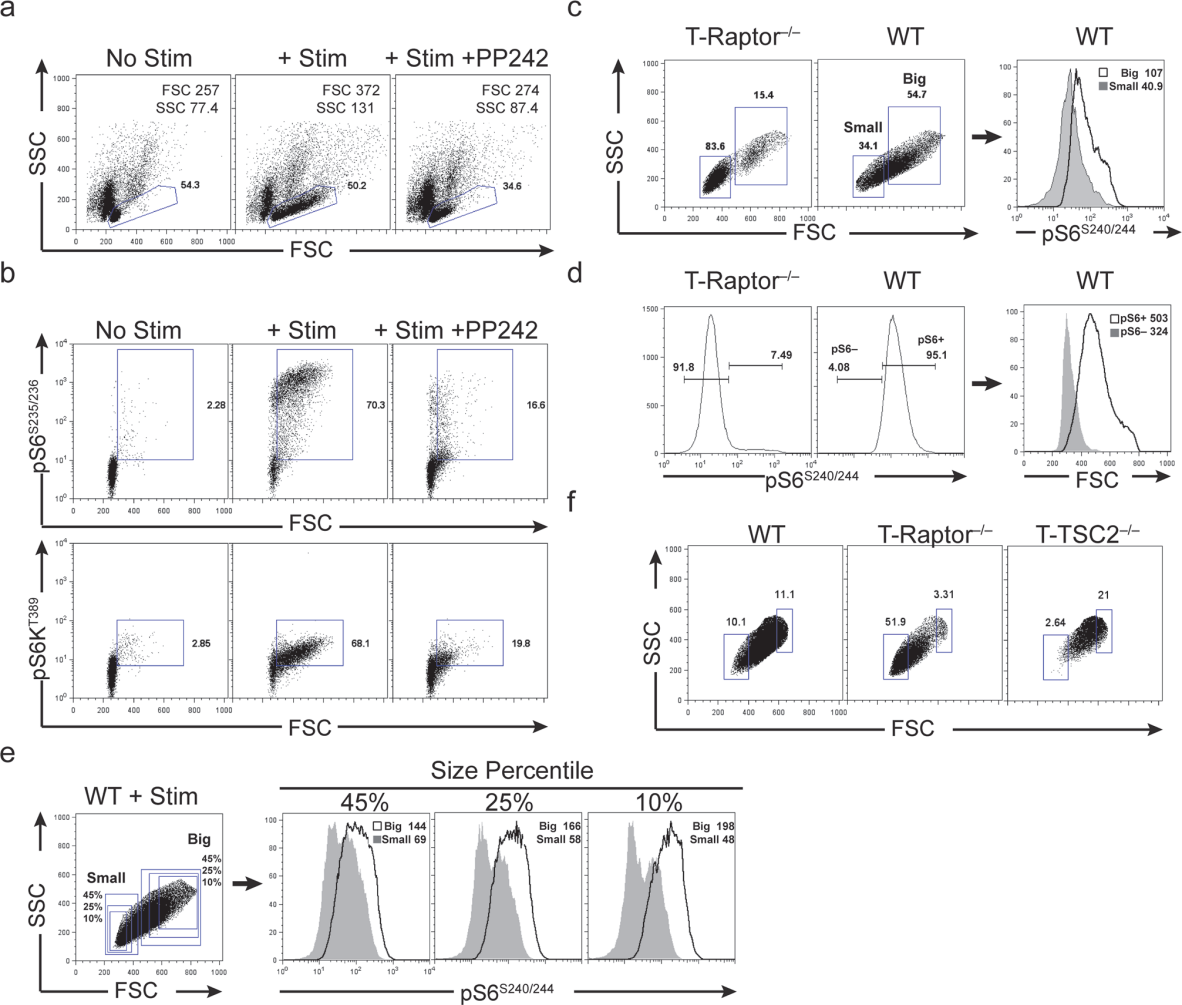
CD4+ T cells coordinately increase in size and mTORC1 activity following TCR stimulation. **a-b)** 5c.c7 Rag^–/—^CD4+ T cells were cultured for 24hrs in media, or media + Pigeon Cytochrome C (PCC) peptide +/- 500nM PP242, a mTOR kinase inhibitor. **a)** Peptide stimulated cells exhibit a significant increase in cell size relative to unstimulated controls. Stimulation-induced cell growth can be inhibited by treatment with PP242. Forward (FSC) and Side scatter (SSC) MFI are shown in top right corner of each panel. **b)** Peptide stimulated CD4+ T cells upregulate mTORC1 activity following TCR stimulation, as indicated by phosphorylation of downstream targets S6 and S6K. This increase in mTOR activity is diminished upon PP242 addition. **c-d)** WT and T-Raptor^–/—^OT-II+ splenocytes were stimulated with OVA^323–339^ for 24hrs. **c)** Gates were set on stimulated CD4+ T cells based upon predominant size of stimulated T-Raptor^–/—^cells and mTORC1 activity was measured from the smallest (Small) and largest (Big) populations of WT stimulated cells. pS6 MFI shown in upper right corner of histogram FACs plot**. d)** Gates were set based upon phosphorylated S6 (pS6) expression detected in T-Raptor^–/—^cells and size (FSC) was assessed based from pS6—and pS6+ populations of WT stimulated cells. FSC MFI shown in upper right corner of histogram FACs plot. **e)** 5c.c7 Rag^–/—^CD4+ T cells were stimulated with PCC for 24hrs and mTORC1 activity (as indicated by pS6 expression) was measured from the 45%, 20%, and 10% smallest (Small) and largest (Big) populations of cells. **f)** WT, T-Raptor^–/—^and T-TSC2^–/—^OT-II+ splenocytes were stimulated with OVA^323–339^ for 24hrs. Plots show percentage of smallest and largest cells in each genotype based on the 10% gate generated from WT stimulated cells. The data are representative of at least 3 independent experiments.

Following TCR stimulation and appropriate co-stimulation, naïve CD4+ T cells dramatically increase in size after 24 hours (**Fig. 1A**). The cellular processes that regulate cell size are in part controlled by mTOR activity [10,26]. To determine if mTOR regulates the size of activated T cells, we stimulated T cells in the presence of mTOR kinase inhibitor, PP242 [27]. TCR-induced blastogenesis is significantly inhibited by the addition PP242 (**Fig. 1A**). Furthermore, T cell activation induces mTORC1 activity as indicated by phosphorylation of ribosomal-S6 at S235/236 and S6 Kinase at T389 (**Fig. 1B**). As expected, phosphorylation of these proteins is diminished with addition of PP242. We consistently find that mTORC1 activity is markedly enriched in the T cells that have increased size (**Fig. 1B**). The increase in size and mTORC1 activity is best observed after 15 hours of stimulation (**S1 Fig.**). Thus, there is a direct correlation of cellular size with mTORC1 activity upon activation of a clonal population of naïve T cells.

To further examine the relationship of mTORC1 activity with cellular size, we examined TCR-induced blastogenesis in Raptor deficient T cells (T-Raptor^–/–^), which have dramatically reduced mTORC1 activity. After 24 hours of stimulation, the vast majority (80%) of the T-Raptor^–/—^T cells remain small in size compared with ~30% of the WT T cells **(Fig. 1C)**. Consistent with the role of mTORC1 in regulating T cell size, the largest cells (labeled ‘Big’) detected after stimulation of WT T cells display the highest mTORC1 activity, while the smallest cells (labeled ‘Small’) demonstrate the lowest mTORC1 activity (**Fig. 1C**). Likewise, WT cells with the highest mTORC1 activity (labeled ‘pS6+’) are the largest in size (**Fig. 1D**). Examination of cell size at the 45^th^, 25^th^ and 10^th^ percentiles reveals a graded effect of mTORC1 activity on cell size, whereby regardless of size percentile, mTORC1 activity is consistently highest in the largest cells (**Fig. 1E**). The largest difference in mTORC1 activity is most evident in the 10% smallest and largest size percentiles (**Fig. 1E**). Thus, we chose to utilize the 10% size cut off in all following experiments to demonstrate the most robust differences between activated cellular populations. This idea was validated by assessing size of WT, T-Raptor^–/–^, and T-TSC2^–/–^(T-TSC2^–/—^T cells have hyperactive mTORC1 activity) OT-II+ splenocytes after stimulation with OVA^323–339^ peptide. Again, we observed that over half of the activated T-Raptor^–/—^CD4+ T cells are detected in the gate set from the 10% smallest activated WT cells, while less than 3% of T-TSC2^–/—^are detected in the same gating strategy (**Fig. 1F**). Based on these results, we propose that cell size can be used as a surrogate indicator of mTORC1 activity in recently stimulated T cells, allowing for easy identification and tracking of both mTOR^hi^ and mTOR^lo^ cells from an initially monoclonal culture of naïve T cells.

We propose that for an individual cell, antigen recognition (Signal 1) will occur in the context of differential mTORC1 activation depending upon accessory signals (Signal 2) from the environment. However, it was critical to demonstrate that the differential activation of mTORC1 was not simply due to lack of antigen recognition. That is, we wanted to ensure that even the cells with decreased mTOR activity still received full antigen receptor engagement.

**Fig 2:**
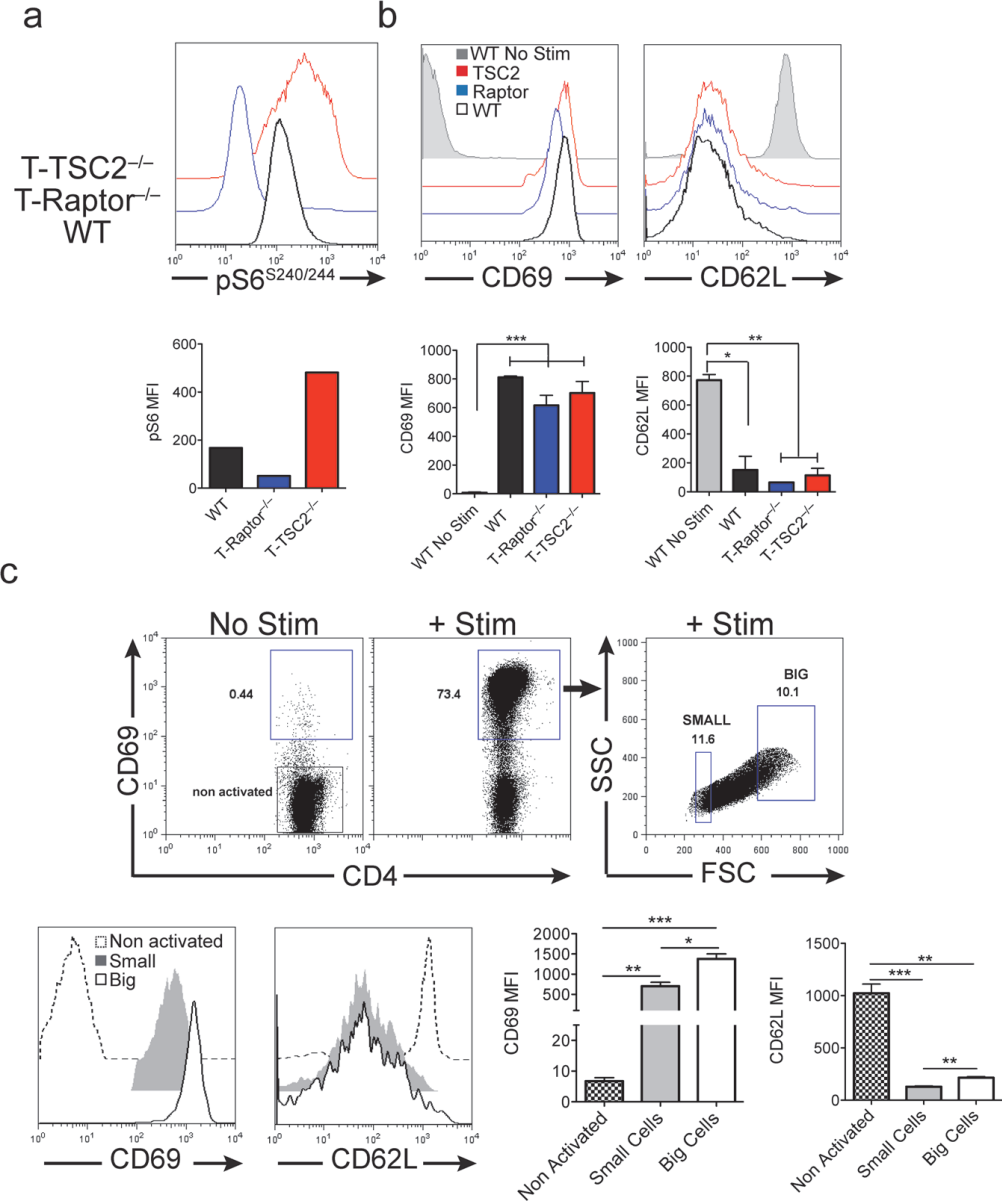
mTORC1 activity is not required for antigenic recognition. **a-b)** WT, T-Raptor^–/—^and T-TSC2^–/—^OT-II+ splenocytes were stimulated with OVA^323–339^ for 24hrs. **a)** mTORC1 activity, as assessed by pS6^S240/244^ expression was measured from the CD4+ population of each genotype. The bar graph below displays the MFI ± standard deviation (S.D.) of pS6 per genotype represented in above histogram plot. **b)** Expression of activation marker CD69, and homing marker, CD62L, are shown from the CD4+ population of each genotype. Below bar graphs indicate MFI of each molecule of interest from 3 independent experiments. **c)** 5c.c7 Rag^–/—^CD4+ T cells were stimulated with PCC for 24hrs and expression of CD69 and CD62L was assessed from the 10% smallest and largest populations of activated CD4+ cells. Expression was compared against non-activated 5c.c7 Rag^–/—^CD4+ T cells (CD4+ CD69–). Bar graphs below represent MFI ± S.D. of each molecule of interest from 3 independent experiments. The data are representative of (a,c) and/or are a composite of 3 independent experiments (b,c).

To this end, WT, T-Raptor^–/—^and T-TSC2^–/—^OT-II^+^ splenocytes were stimulated with OVA^323–339^ peptide for 24 hours. As expected, this led to differential activation of mTORC1, whereby T-TSC2^–/—^T cells have increased activity while T-Raptor^–/—^T cells have diminished activity compared to WT cells (**Fig. 2A**). Notably, there was robust and roughly equivalent upregulation of CD69 and downregulation of CD62L in T cells from all 3 genotypes (**Fig. 2B**). That is, diminished or enhanced mTORC1 activity did not affect antigen-induced activation. Next, we wanted to demonstrate that such was also the case for the small (mTOR^lo^) and big (mTOR^hi^) populations of WT cells. Splenocytes from 5c.c7 Rag^–/—^TCR transgenic mice were stimulated with Pigeon Cytochrome C (PCC) peptide. Upon stimulation, there is robust activation as indicated by CD69 upregulation (**Fig. 2C**). Starting from this clonotypic population of naïve T cells, we observe that there emerges a heterogeneous population of antigen experienced cells that differ dramatically in size. Importantly, this difference in cell size was not due to a lack of activation as both large and small cells express high levels of the early T cell activation marker CD69, and demonstrate robust downregulation of CD62L relative to naïve T cells (**Fig. 2C**). We did note, however, that the levels of CD69 and CD62L were slightly decreased in the T-Raptor^–/—^and mTOR^lo^ cells when compared to WT and mTOR^hi^ T cells (Fig. 2B and 2C). The significance of this finding is unclear.

### mTOR^hi^ and mTOR^lo^ CD4+ T cells display differential proliferative, metabolic and survival fates

**Fig 3:**
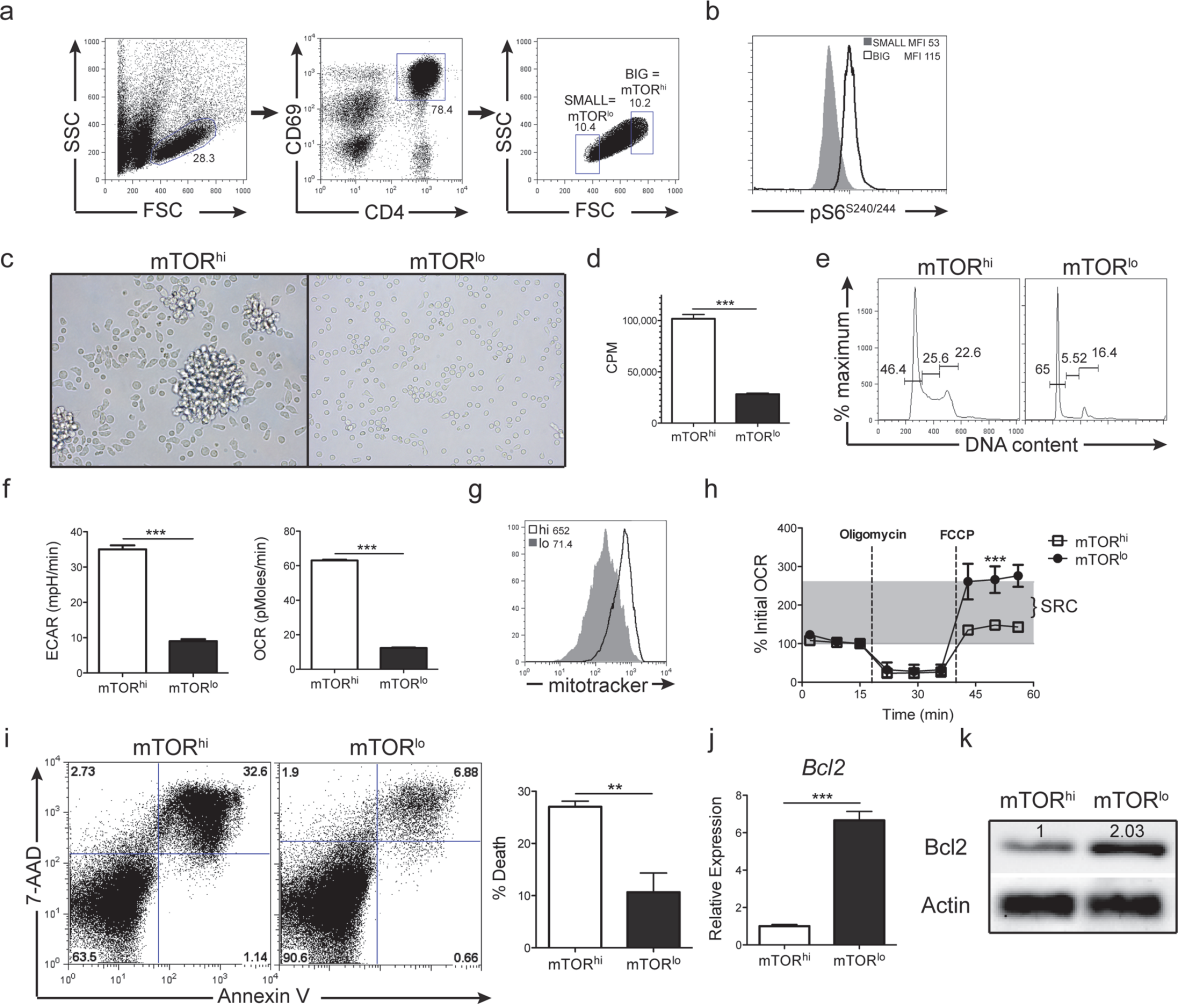
Cell size can be used to isolate recently stimulated CD4+ T cells populations with distinct proliferative, metabolic and survival profiles. **a)** Splenocytes from 5c.c7 Rag^–/—^mice were stimulated with 0.5ug/ml PCC peptide for 24hrs then sorted into CD4+CD69+ populations with FSC/SSC “big” mTOR^hi^ or FSC/SSC “small” mTOR^lo^ profiles. **b)** mTORC1 activity was assessed from “Big” and “Small” cells immediately after sorting. pS6 MFI is shown in the upper corner of the plot. **c)** Sorted mTOR^hi^ cells form large homotypic aggregates when cultured in media containing IL-2 for 4hrs after sorting. **d-h)** Sorted cells were cultured in media containing IL-2 for 24hrs. **d)** mTOR^hi^ cells exhibit a higher proliferative rate—as measured by ^3^H-thymidine incorporation—than their mTOR^lo^ counterparts. **e)** Sorted mTOR^hi^ cells show robust cell cycle progression 24hrs post sorting, while sorted mTOR^lo^ cells arrest in the G_0/1_ stage. **f)** mTOR^hi^ cells display a significantly higher Extracellular Acidification Rate (ECAR) and Oxygen Consumption Rate (OCR) than their sorted mTOR^lo^ counterparts. The increased metabolic activity observed in the sorted mTOR^hi^ cells is accompanied by a higher mitochondrial load than sorted mTOR^lo^ cells as indicated by mitotracker green staining (**g**). Mitotracker green MFI shown in upper corner of FACs plot. **h**) Sorted mTOR^lo^ CD4+ T cells exhibit a significantly higher Spare Respiratory Capacity (SRC) than sorted mTOR^hi^ cells. **i)** Sorted mTOR^hi^ cells exhibit a higher rate of cell death when cultured for 5 days in media supplemented with IL-2 than sorted mTOR^lo^ cells. The bar graph to the right depicts % AnnexinV+7-AAD+ cells ± S.D. from 3 independent experiments. Three days after culture in media + IL-2, sorted mTOR^lo^ cells show significantly higher levels of (**j**) Bcl-2 mRNA and (**k**) protein expression than sorted mTOR^hi^ cells. mRNA transcript expression shows mean relative expression ± S.D. from 3 independent experiments, with the mTOR^hi^ values set to 1. Bcl-2 protein expression was normalized to Actin and fold difference in expression is shown above the protein band. The data are representative of at least 3 independent experiments.

In light of the relationship we observed between cell size and mTOR activity in recently activated CD4+ T cells, we sought to track the fate of T cells with differing levels of mTOR activity, as indicated by cell size, following antigen recognition. To this end, naïve 5c.c7 TCR transgenic Rag^–/—^splenocytes were stimulated with PCC peptide for 24 hours. To ensure that only stimulated CD4+ T cell were included in future analysis, cells were stained for CD4 and the mTOR independent early activation marker CD69, then sorted into CD4+CD69+ FSC/SSC “big” mTOR^hi^ and CD4+CD69+ FSC/SSC “small” mTOR^lo^ populations (**Fig. 3A**). To further confirm the relationship of mTORC1 activity with cell size, sorted cells were immediately assessed for mTORC1 activity. As expected, the largest population (labeled ‘Big’) cells demonstrate the highest phosphorylated S6 (pS6) expression (**Fig. 3B**). At the time of sorting, the process of cellular division had not yet been initiated, with ~90% of both the mTOR^hi^ and mTOR^lo^ populations still in G_0_ stage of the cell cycle as indicated by propidium iodide staining (**S2A Fig.**). After the sort, cells were cultured in media supplemented with 1ng/ml IL-2. Sorted mTOR^hi^ cells rapidly form large homotypic aggregates in culture after 4 hours post sorting, which did not occur in sorted mTOR^lo^ cultures (**Fig. 3C**). Twenty four hours after culture, sorted mTOR^hi^ and mTOR^lo^ cells were treated with tritiated (^3^H-) thymidine to monitor proliferation and cultured for an additional 16 hours. Sorted mTOR^hi^ CD4+ T cells exhibit a much higher rate of proliferation than sorted mTOR^lo^ cells, as indicated by ^3^H-thymidine uptake (**Fig. 3D**). Additionally, CD4+ mTOR^hi^ cells display robust cell cycle progression relative to sorted mTOR^lo^ cells, which arrest in G_0/1_ following the sort (**Fig. 3E**). CFSE labeling of 5c.c7 splenocytes prior to stimulation confirmed that neither mTOR^hi^ and mTOR^lo^ sorted cellular populations have initiated division at the time of sorting, but mTOR^hi^ cells have enhanced proliferation as indicated by CFSE dilution after 3 days of culture in IL-2 supplemented media (**S2B Fig.**).

mTOR plays an important role in the regulation of cellular metabolism [13,28]. As such, we next assessed the metabolic profile of the cells. Sorted mTOR^hi^ cells are highly metabolically active, exhibiting a significantly higher Extracellular Acidification Rate (ECAR)—an indirect indicator of glycolysis—than their mTOR^lo^ counterparts (**Fig. 3F)**. In addition, the mTOR^hi^ cells initially demonstrate a higher Oxygen Consumption rate (OCR) compared to mTOR^lo^ cells (**Fig. 3F**). This higher rate of metabolic activity observed in the sorted mTOR^hi^ cells is accompanied by an increase in mitochondria content (**Fig. 3G**). The higher ECAR observed in sorted mTOR^hi^ CD4+ T cells is consistent with the role of mTOR in promoting glycolysis [28]. Further, the decreased ECAR observed in the small, mTOR^lo^ cells phenocopies the metabolic profile of rapamycin treated CD4+ T cells (**S3A Fig.**). Thus, within a clonal population of T cells stimulated with their cognate antigen, those cells with high levels of mTORC1 activity demonstrate increased proliferation and metabolic activity when compared to those cells with relatively low mTORC1 activity.

We have shown that sorted mTOR^lo^ CD4+ T cells exhibit a markedly decreased glycolytic metabolic profile than sorted mTOR^hi^ cells. However, sorted mTOR^lo^ cells possess increased Spare Respiratory Capacity (SRC) when compared to mTOR^hi^ cells from the same culture (**Fig. 3H**). A similar finding was also observed for CD4+ T cell stimulated in the presence of rapamycin (**S3B Fig.**). SRC is calculated as the differential between a cell’s basal rate of oxygen consumption and the maximal rate of oxygen consumption. SRC is a metric indicating how close a cell is currently functioning to its maximal metabolic potential. Rapidly proliferating cells, such as terminally differentiated effector T cells and tumor cells, display a very low SRC [29,30]. Conversely, long-lived cells, such as stem cells and memory T cells have been shown to have a large SRC [29]. This correlation between longevity and SRC is thought to be due to the need for long-lived cells to survive periods of metabolic stress, which would prove fatal to more terminally differentiated cell types. In light of the previously described association between SRC and longevity, we assayed the *in vitro* survival of sorted mTOR^hi^ and mTOR^lo^ CD4+ T cells. From a clonal population of activated T cells, the mTOR^hi^ cells undergo increased apoptosis compared to sorted mTOR^lo^ counterparts when cultured in media supplemented with IL-2 for 5 days after sorting (**Fig. 3I**). The increased survival observed in the mTOR^lo^ cells was accompanied by significantly higher expression of the anti-apoptotic protein Bcl-2 at both a transcriptional (**Fig. 3J**) and protein **(Fig. 3K**) level.

### mTOR^lo^ cells express increased levels of CD25

**Fig 4:**
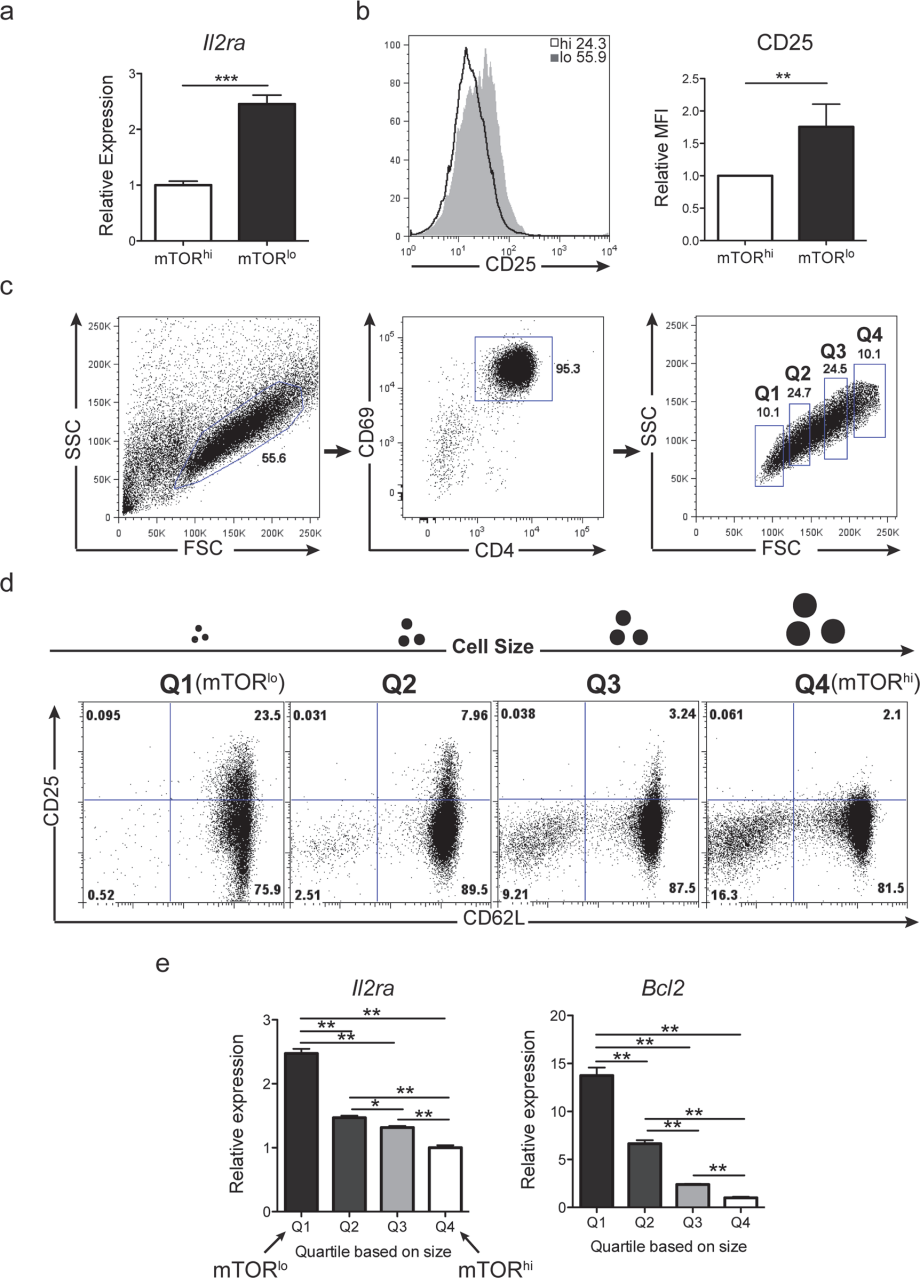
Divergent fates of activated CD4+ T cells can be tracked with gradients of cellular size following activation. **a-b)** Splenocytes from 5c.c7 Rag^–/—^mice were stimulated with 0.5ug/ml PCC peptide for 24hrs and then sorted into CD4+CD69+ populations with FSC/SSC “big” mTOR^hi^ or FSC/SSC “small” mTOR^lo^ profiles. Cells were cultured in media supplemented with IL-2 for 3 days. **a)** 3 days after sort, mTOR^lo^ cells express higher mRNA transcript, and **b)** surface protein expression of CD25 than sorted mTOR^hi^ cells. The bar graph to the right depicts the fold increase in CD25 MFI expression in mTOR^lo^ cells compared to mTOR^hi^ cells from 6 replicate experiments, with expression of mTOR^hi^ cells set to a value of 1. Error bars represent ± S.D, n = 3 (a) or n = 6 (b). **c-d)** 5c.c7 Rag^–/—^splenocytes were stimulated with PCC for 24hrs and activated (CD69+) CD4+ cells were sorted based on 4 size profiles. Cells were sorted into 10% smallest (Q1) and largest (Q4) populations in addition to the next 25% smallest (Q2) and largest (Q3) populations. **c)** Representation of sorting scheme utilized. **d)** After the sort, cells were cultured separately in IL-2 supplemented media for 3 days. On day 3, expression of CD25 and CD62L was assessed by flow cytometry. An illustration of cell size after sort is depicted above flow plots for clarification of sorted quartile populations. **e)** Purified CD4+ T cells from 5c.c7 Rag^–/—^mice were stimulated by plate bound anti-CD3 and anti-CD28 for 24hrs. Cells were sorted as indicated in **c,** and mRNA transcript expression of CD25 and Bcl-2 was determined from sorted populations after a 3 day culture in IL-2 supplemented media. Bar graphs show relative expression ± S.D. from 3 independent experiments. The data are representative of 3 independent experiments or show cumulative results.

While mTORC1 activity is reduced in the sorted small cells compared to big cell counterparts, Bcl-2 levels are highest in small, mTOR^lo^ cells. Many factors have been shown to control Bcl-2 expression in CD4+ T cells, including cytokine receptor signaling [31]. To this end, we tested for expression of the high affinity IL-2 receptor CD25 in our sorted populations. Interestingly, CD25 expression at the transcript and protein level was higher on the mTOR^lo^ cells (Fig. 4A and 4B). To determine if this phenotype was graded across a spectrum of cell size, we analyzed CD69+ T cells from 4 distinctly sized populations: the 10% smallest (Q1: mTOR^lo^), 10% largest (Q4: mTOR^hi^) populations as previously assessed, in addition to the next 25% smallest (Q2) or largest (Q3) groups of cells (**Fig. 4C**). These studies revealed that CD25 protein and transcript levels, CD62L protein expression, and Bcl-2 transcript levels are inversely related to cell size and hence mTORC1 activity (Figs. 4D and 4E **and** S4A). Thus, from a metabolic and survival perspective, antigen recognition in the setting of strong mTORC1 activation generates cells with increased glycolytic flux that are destined to be short-lived. Alternatively, antigen recognition in the context of decreased mTORC1 activation leads to the generation of long-lived cells with increased SRC, increased Bcl-2 and increased CD25 (IL-2Ra) expression.

### Short-lived, metabolically active, mTOR^hi^ cells demonstrate an effector phenotype

**Fig 5:**
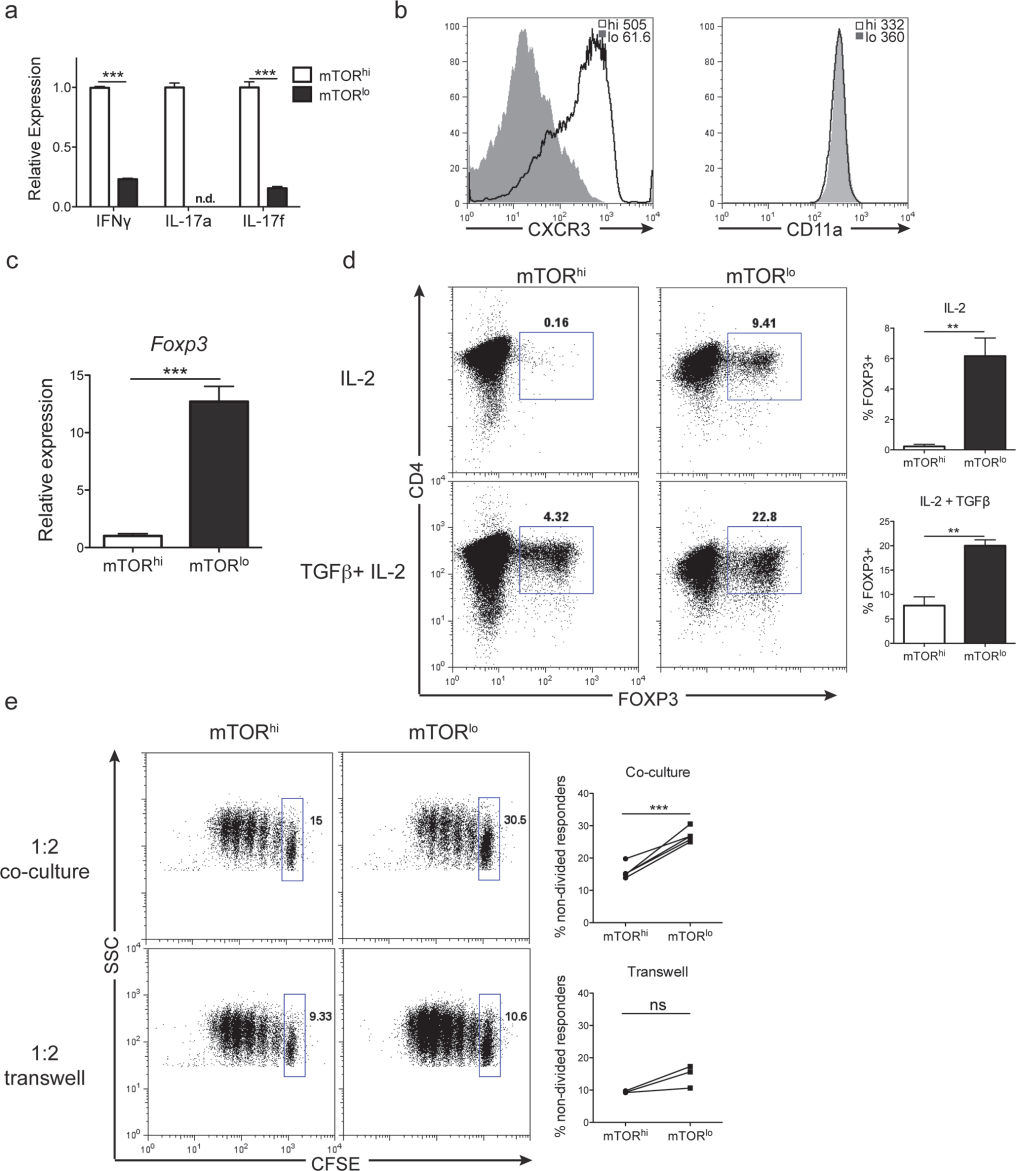
Cell size can be used to isolate recently stimulated CD4+ T cell populations with distinct immunological fates. **a)** Relative expression of IFN-gamma, IL-17a and IL-17f mRNA transcripts were determined from mTOR^hi^ and mTOR^lo^ populations immediately after sorting stimulated 5c.c7 Rag^–/—^splenocytes. Error bars show ± S.D. from 3 independent experiments, and mTOR^hi^ values were set to 1. **b-d**) Sorted populations of cells were cultured in media supplemented with IL-2 for 3 days prior to assessment. **b**) Expression of chemokine receptor, CXCR3, and LFA-1 subunit CD11a was determined by flow cytometry. MFI per population is shown at the top of each FACs plot. **c**) Relative expression of Foxp3 mRNA transcript ± S.D. was determined from the mTOR^lo^ population compared to the mTOR^hi^ cells. The graph was generated from 3 independent experiments, and mTOR^hi^ values were set to 1. **d)** Foxp3 protein expression was determined from sorted mTOR^lo^ and mTOR^hi^ populations 3 days after culture in IL-2 alone or IL-2 + TGF-ß. Bar graphs to the right depict % Foxp3+ cells ± S.D. generated in each population from 3 independent experiments. **e**) mTOR^lo^ Foxp3+ regulatory T cells possess the ability to suppress the proliferation of naïve CD4+ T cells. Sorted mTOR^hi^ or mTOR^lo^ populations were cultured in media supplemented with IL-2 for 3 days prior to use in the suppression assay. The percentage of non-divided responders was determined by CFSE dilution 72hrs after culture of 1:2 suppressors: responders in direct contact or in transwell suppression assays. The graphs to the right depict the percentage of non-divided responders after culture with mTOR^hi^ or mTOR^lo^ ‘suppressors’ from 3 independent experiments. Connecting lines show values from sorted populations from the same experiment. The data are representative of at least 3 independent experiments or show cumulative results.

Our data thus far demonstrate that for a population of clonal T cells, metabolic and survival potential is dictated by their level of mTOR activity after activation. Within this context, we hypothesized that the short-lived mTOR^hi^ cells represent the activated cells destined to become effector cells. Consistent with this hypothesis, recently stimulated and sorted mTOR^hi^ CD4+ T cells express significantly higher levels of IFN-gamma, IL-17a and IL-17f transcript than sorted mTOR^lo^ cells (**Fig. 5A**). Additionally, sorted mTOR^hi^ cells express much higher cell surface levels of the chemokine receptor CXCR3 (associated with pro-inflammatory effector cells) [32] than sorted mTOR^lo^ cells (**Fig. 5B**). As a negative control, no difference was observed in the expression of the LFA-1 subunit CD11a.

Upon antigen recognition, mTOR^lo^ cells demonstrate metabolic programs consistent with long-lived cells. However, we have previously demonstrated that TCR engagement in the absence of mTOR induces the generation of Foxp3+ regulatory T cells [14]. Furthermore, others have shown that T cell activation in the presence of the mTOR inhibitor rapamycin promotes the generation of Foxp3+ regulatory T cells [15–18]. Therefore, we wondered if a sub-population of the mTOR^lo^ cells might be Foxp3+ regulatory T cells. Consistent with this hypothesis, we observed that the mTOR^lo^ population was enriched for Foxp3+ T cells (Fig. 5C and 5D). That is, under normally activating culture conditions (antigen activation followed by IL-2 addition), 9.4% of the mTOR^lo^ cells express Foxp3, while only 0.16% of the mTOR^hi^ cells expressed Foxp3 (**Fig. 5D**). This difference was also observed and was amplified when TGF-ß was added to the cultures (22.8% versus 4.3%). Foxp3 expression is upregulated in every division of activated mTOR^lo^ cells after culture in IL-2, and a graded decrease in Foxp3 expression is observed in each population of increased cell size/mTORC1 activity (**S4B Fig.**). The preferential induction of Foxp3 expression in sorted mTOR^lo^ cells is not limited to cultures derived from homogeneous Rag^–/—^TCR transgenic splenocytes, but can also be observed in cultures generated from anti-CD3 stimulated polyclonal WT CD4+ T cells (**S5A Fig.)**. Not unexpectedly, the mTOR^lo^ CD4+ T cells cultured in IL-2 alone were better able to suppress the proliferation of activated CD4+ T cells compared to their cultured mTOR^hi^ counterparts (**Fig. 5E**). This enhanced suppression was most likely due to the increase of Foxp3+ T cells generated from the mTOR^lo^ sorted population compared to the mTOR^hi^ cells (**S5B Fig.**). The ability of the mTOR^lo^ T cells to suppress responder T cells was markedly diminished when the responders and suppressors were separated in a transwell, indicating that the suppression is primarily contact dependent (**Fig. 5E**).

A question remained if Foxp3+ T cells were generated *de novo* from the activated mTOR^lo^ cells, or if the enhanced generation of Foxp3+ T cells observed in the sorted mTOR^lo^ populations was simply a consequence of sorting on a high frequency of already established natural Tregs. In order to answer this question, we utilized mice which express green fluorescent protein (GFP) under the control of the Foxp3 promoter (Foxp3^GFP+^) [33]. Splenocytes from WT Foxp3^GFP+^ mice were stimulated with 0.1ug/ml anti-CD3 for 20 hrs. Activated (CD69+) CD4+ GFP—cells were sorted from the 10% biggest ‘mTOR^hi^’ and smallest ‘mTOR^lo^’ populations (**S6A Fig.**). This approach ensured that all GFP+ natural Tregs would be removed from our sorted populations. Phenotypic assessment of sorted mTOR^hi^ and mTOR^lo^ populations 72 hrs after culture in IL-2 revealed that suppressive Foxp3+ T cells were generated *de novo* from the *in vitro* stimulated splenocytes (S6B–C Fig **.**). These observations were consistent with our previous results demonstrating that mTOR^lo^ CD4+ T cells have a greater propensity to differentiate into Foxp3+ T cells (Fig. 5C–5E).

**Fig 6:**
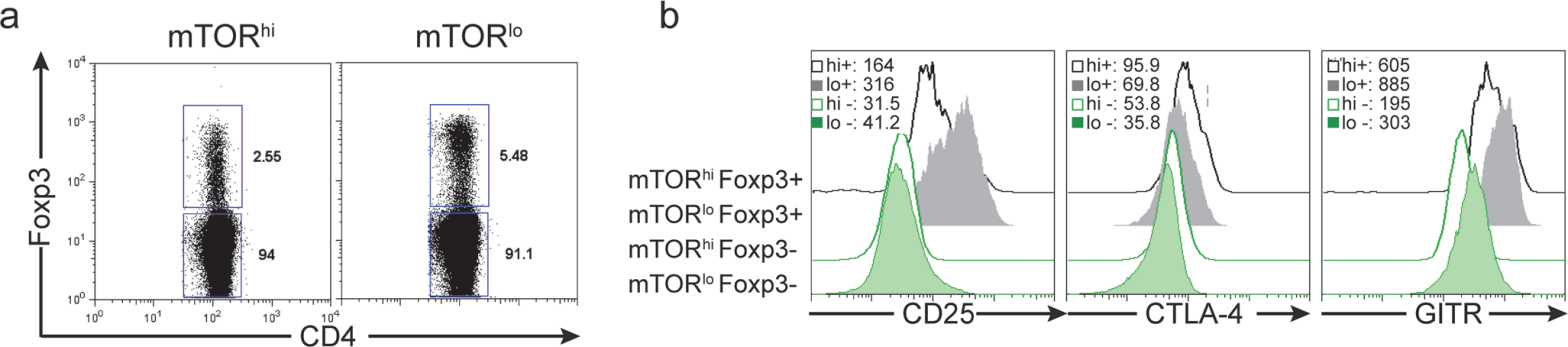
Foxp3+ CD4+ T cells generated from mTOR^hi^ and mTOR^lo^ sorted populations have distinct phenotypes. Splenocytes from 5c.c7 Rag^–/—^TCR transgenic mice were stimulated with 0.5ug/ml PCC peptide for 24hrs, and then sorted into CD4+CD69+ populations with FSC/SSC “big” mTOR^hi^ or FSC/SSC “small” mTOR^lo^ profiles. Cells were cultured in media supplemented with IL-2 for 96hrs, and analyzed by flow cytometry. **a)** The percentage of CD4+ Foxp3+ cells was determined from each population. **b)** Histograms show shifts in MFI of markers associated with a T-reg phenotype. Populations were gated from the Foxp3+ or Foxp3- cells of the mTOR^hi^ or mTOR^lo^ sorted samples. The MFI of each protein is shown in the top corner of each FACs plot. The data are representative of at least 3 independent experiments.

Our data thus far suggests that under normally activating conditions, the recognition of antigen in the context of low mTOR activation promotes the *de novo* generation of regulatory T cells. Nonetheless, we could still detect a small percentage of Foxp3+ T cells which displayed high mTOR activity. Therefore, we sought to further phenotype the Foxp3+ T cells generated from the sorted mTOR^lo^ and mTOR^hi^ populations. First, Foxp3+ T cells derived from either mTOR^lo^ and mTOR^hi^ populations have higher expression of the known T-regulatory cell markers, CD25, CTLA-4, and GITR when compared to Foxp3 negative counterparts (Fig. 6A and 6B). Interestingly, Foxp3+ mTOR^lo^ T cells have enhanced expression of CD25 and GITR compared to mTOR^hi^ derived Foxp3+ cells (**Fig. 6B**). Alternatively, Foxp3+ mTOR^hi^ T cells have enhanced expression of CTLA-4 compared to Foxp3+ cells derived from the mTOR^lo^ population (**Figs.** 6B **and** S5). Thus, while the generation of Foxp3+ T cells is clearly enriched in the mTOR^lo^ population of antigen-activated T cells, there also exists a small but distinct population of Foxp3+ CTLA-4^hi^ T cells which emerges from the mTOR^hi^ population of antigen activated cells.

### Divergent phenotypic properties of mTOR^hi^ and mTOR^lo^ Foxp3+ regulatory T cells

**Fig 7:**
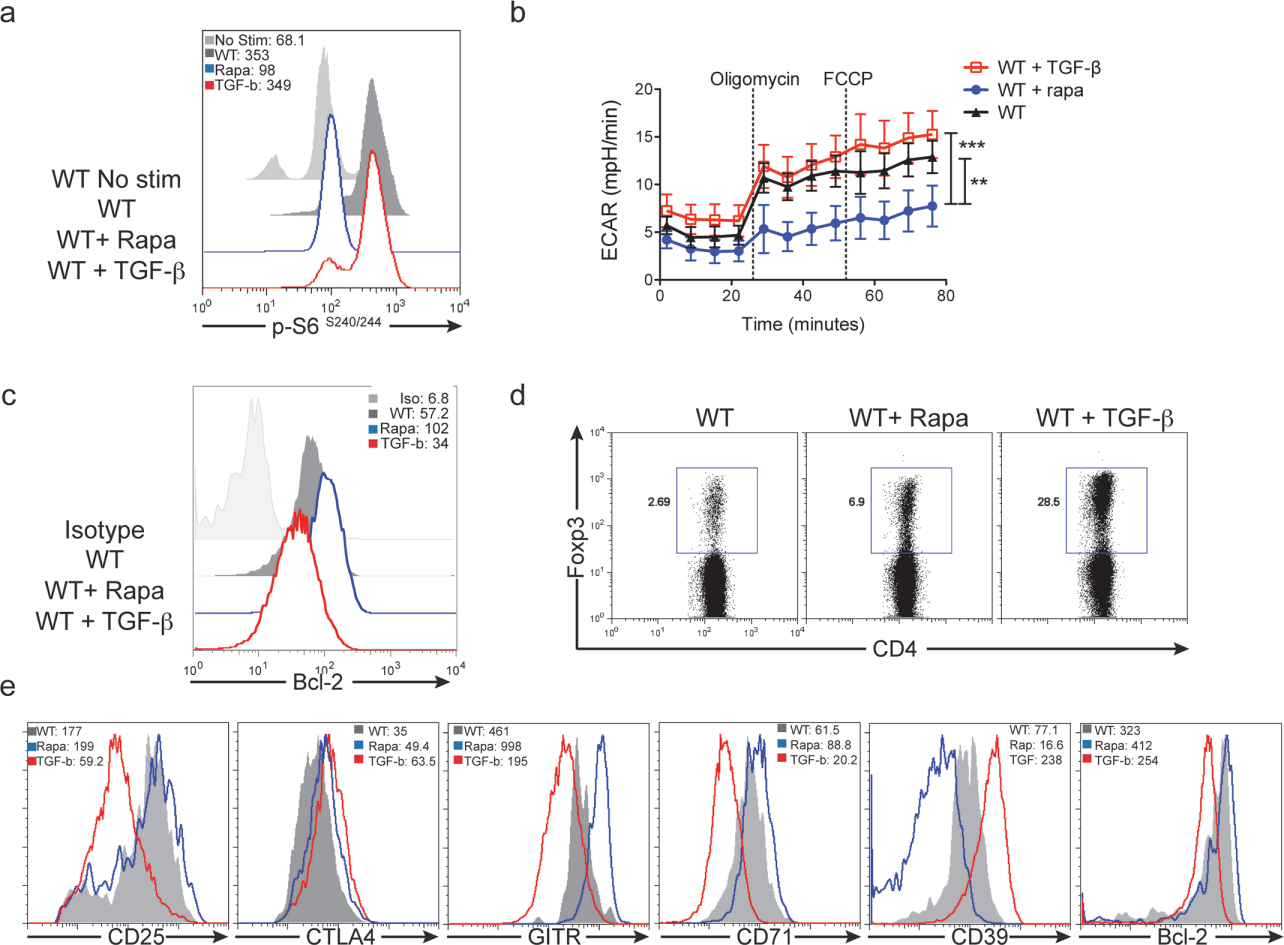
Foxp3+ CD4+ regulatory T cells exhibit divergent phenotypic profiles, dependent on their method of induction. Splenocytes from 5c.c7 Rag^–/—^TCR transgenic mice (**a**) or WT C57BL/6 mice (**b-e**) were stimulated with 1ug/ml soluble anti-CD3 and 1ng/ml IL-2 in the presence or absence of either 10ng/ml TGF-ß or 500nM rapamycin for 24hrs. **a**) mTORC1 activity was measured by flow cytometric analysis of phosphorylated-S6 expression 24hrs after stimulation. Histograms were gated from activated CD4+CD69+ populations within each culture condition. **b-e)** After 24hrs of stimulation, cells were expanded in media supplemented with IL-2 alone, IL-2 + TGF-ß, or IL-2 + rapamycin for an additional 72hrs before phenotypic assessment. **b)** Cells were run on an extracellular flux analyzer and the extra cellular acidification rate, ECAR, was determined over time. **c)** Bcl-2 expression and **d**) Foxp3 expression levels were assessed from CD4+ T cells of each culture condition. **e)** Histograms show shifts in MFI of markers associated with a T-reg phenotype. Populations were gated from Foxp3+ CD4+ T cells. The MFI of each population is shown in the upper corners of the FACS plots depicted in a,c,and e. The data are representative of 3 independent experiments.

Our data are consistent with previous findings that diminished mTOR activity in T cells promotes the generation of regulatory T cells [14–18]. However, we also find a small, but distinct population of Foxp3+ T cells generated from mTOR^hi^ cells. Interestingly, one of the most common techniques to generate regulatory T cells *in vitro* is to induce robust activation in the presence of high concentrations of TGF-ß [34]. In fact, stimulation under these conditions results in robust mTOR activation (**Figs.** 7A **and** S7). That is, while strong TCR engagement in the presence of TGF-ß promotes the generation of Foxp3+ T cells, it also results in robust mTORC1 activation. Given the highly divergent metabolic profiles and survival potentials of sorted mTOR^lo^ and mTOR^hi^ conventional CD4+ T cells, we wanted to determine if Foxp3+ CD4+ T cells generated in the presence of high or low mTOR activity during TCR stimulation demonstrate similar metabolic and survival potentials. To this end, WT splenocytes were stimulated with 1ug/ml anti-CD3 and 1ng/ml IL-2 in the presence or absence of either 10ng/ml TGF-ß or 500nM rapamycin. Anti-CD3 was washed out at 24 hrs, and the cultures were expanded in media supplemented with IL-2 alone, IL-2 + TGF-ß, or IL-2 + rapamycin for 72 hrs before harvest for flow-cytometric analysis. As expected, the Foxp3+ T cells from the rapamycin treated cultures had reduced mTORC1 activity (mTOR^lo^) compared to the Foxp3+ T cells generated in the presence of TGF-ß (**Fig. 7A**). As was the case for the mTOR^hi^ CD4+ sorted T cell populations, CD4+ T cells cultured with TGF-ß demonstrate enhanced ECAR compared to the cells activated in the rapamycin treated conditions (**Fig. 7B**), but reduced expression of Bcl-2 (**Fig. 7C**). These data suggest that culture in TGF-ß promotes an ‘effector’ phenotype with increased glycolysis but diminished survival. Activation of T cells in the presence of rapamycin or TGF-ß enhanced Foxp3+ T reg generation compared to untreated, activated cells (**Fig. 7D**), and we further assessed the phenotype of Foxp3+ cells generated from these culture conditions (**Fig. 7E**). Notably, phenotypic analysis of the Foxp3+ cells generated from the rapamycin versus TGF-ß culture conditions recapitulated the results observed from the sorted mTOR^lo^ and mTOR^hi^ Foxp3+ populations. Foxp3+ T cells generated in the presence of rapamycin have enhanced expression of CD25, GITR, CD71, and Bcl-2 compared to Foxp3+ T cells derived from TGF-ß cultures (**Fig. 7E**). Alternatively, Foxp3+ cells generated in the presence of TGF-ß have enhanced CTLA4 and CD39 expression compared to those generated in rapamycin (**Fig. 7E**). These data suggest that regulatory T cells generated in the presence of robust mTOR activity (by culture in high concentrations of TGF-ß) have differential survival potential and are metabolically distinct from those generated in the setting of low mTOR activity.

### Differential fates of mTOR^hi^ and mTOR^lo^ human CD4+ T cells

**Fig 8:**
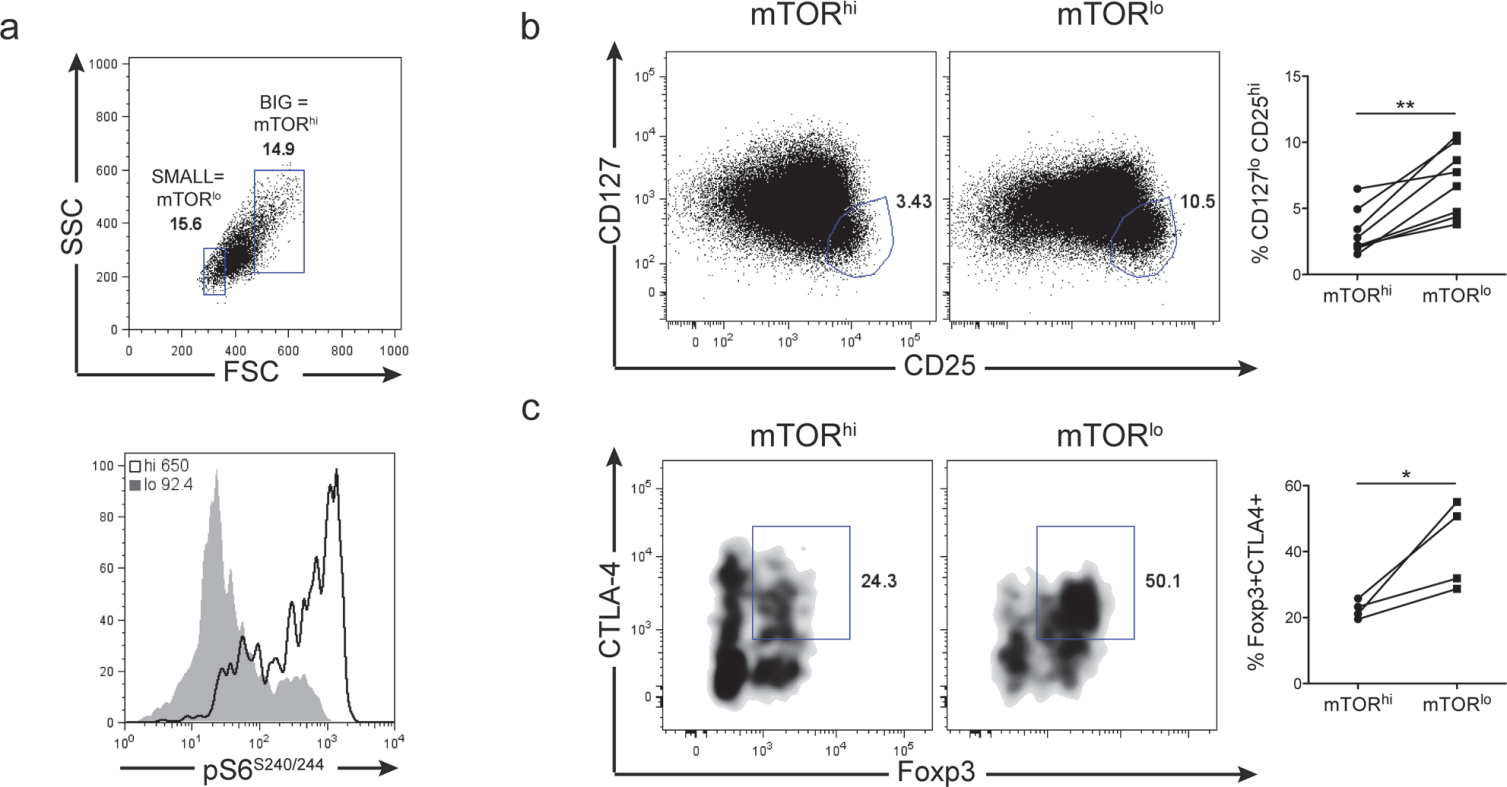
Cellular size (indicative of mTORC1 activity) influences the phenotype of human CD4+ T cells similar to mouse. CD4+ T cells were purified from the PBMCs of 8 human volunteers. Cells were stimulated with human anti-CD3 and anti-CD28 activator beads overnight. **a)** 24hrs after stimulation, mTORC1 activity was assessed by the pS6 expression of the 15% largest and smallest activated (HLA-DR+) CD4+ T cells. Histograms in the lower FACs plot were determined from the ‘big’ and ‘small’ gates shown in the upper plot. The pS6 MFI is shown in the upper corner of the FACs plot. **b-c)** 24hrs after stimulation, the cells were sorted from the 15% largest and smallest activated (CD38+) CD4+ T cells. Sorted populations were cultured in media supplemented with human IL-2 for 72 hrs. After 72 hrs, the phenotype of the large ‘mTOR^hi^’ and small ‘mTOR^lo^’ populations was assessed by flow cytometry. **b)** Human T-reg populations were determined from the CD127^lo^ CD25^hi^ gating strategy. **c**) FACs plots show percentage of Foxp3+ CTLA-4+ double positive cells gated from the ‘T-reg’ gate in b. Statistics are shown to the right. The connecting lines demonstrate the changes in percentages between the mTOR^hi^ and mTOR^lo^ sorted populations from the same individual. The data are representative of 8 distinct human samples.

Next, we wanted to determine if we could use cell size/mTOR activity to track the fate of TCR-activated human T cells. CD4+ T cells from fresh PBMCs were isolated and the T cells were activated with human anti-CD3/anti-CD28 activator beads. Similar to our observations employing murine T cells, mTORC1 activation correlates with cellular size in activated human CD4+ T cells (**Fig. 8A**). Upon sorting and expanding activated mTOR^hi^ and mTOR^lo^ T cells for 72 hrs in IL-2, mTOR^lo^ T cells have an increase in percentage of CD127^lo^CD25^hi^ CD4+ T cells compared to mTOR^hi^ counterparts (**Fig. 8B**). Furthermore, from the CD127^lo^CD25^hi^ population, there is an increase in Foxp3+ CTLA4+ T cells detected in the mTOR^lo^ T cell cultures (**Fig. 8C**). Thus, similar to murine T cells, TCR-stimulation of human CD4+ T cells in the presence of low mTORC1 activity enhances T reg cell differentiation.

## Discussion

We have observed that within a clonal population of newly activated T cells there exists significant heterogeneity of mTORC1 activity. This heterogeneity leads to cells with differential metabolic programs, survival potential and ultimately phenotypic fate. Our data demonstrate that cell size can be utilized as a surrogate indicator of mTOR activity in recently stimulated CD4+ T cells. This observation provides a simple tool allowing for the isolation of T cell populations with distinct metabolic, proliferative, immunologic and survival potential from a heterogeneous population of activated cells.

We demonstrate a direct correlation between the level of mTOR activity upon antigen recognition by CD4+ T cells and the regulation of metabolism and survival. In part, this appears to be mediated by differential expression of anti-apoptotic members of the Bcl-2 protein family facilitated by expression of pro-survival cytokine receptor CD25. While mTOR activity is strongly induced by many pro-survival growth factors such as IL-2 [11], long term cellular survival necessitates the adoption of a quiescent metabolic and proliferative phenotype [13,35]. For example, T cells lacking the ability to enter a state of quiescence due to genetic deletion of the mTOR inhibitor TSC1 are incapable of long term survival *in vivo* [36]. A unique metabolic feature of long-lived stem cells and memory T cells is the presence of an increased SRC [29]. Indeed, our data demonstrate that the mTOR^lo^ T cells possess both a greater SRC (**Fig. 3H**), and survival potential than mTOR^hi^ T cells (Figs. 3I–3K). In this context, it is interesting to note that decreased mTOR activity has not only been associated with increased cellular survival but also with increased longevity at the level of the whole organism [37].

We postulate that there are many factors that contribute to the heterogeneous activation of mTORC1 upon antigen recognition. For example, TCR signal strength has been shown to be important for the polarization of effector lineages in CD4+ and CD8+ T cells [38–41]. While TCR avidity was the same in our clonal population of 5c.c7 T cells, the observed modulation in mTORC1 signaling may be due to competition for peptide on the APCs, the relative activation status of a particular APC, the relative upregulation and encounter with inhibitory ligands and even the local exposure to various activating and inhibitory cytokines. Indeed, mTOR is activated by and thus integrates multiple inputs from the immune microenvironment, which in turn influence the fate of an individual cell’s encounter with antigen [42].

Previous studies have demonstrated the differences in metabolic function between effector and memory CD8+ T cells [29,43,44]. That is, effector CD8+ T cells are shown to be highly glycolytic while memory CD8+ T cells are less glycolytic and demonstrate increased SRC. Likewise, several studies have demonstrated the ability of the mTOR inhibitor rapamycin to promote the generation of CD8+ memory T cells [21,23]. Our current studies employing CD4+ T cells are supported by these previous observations. Our ability to track cells based on the level of mTOR activation has enabled us to link mTOR activity with the metabolic and survival programs necessary to support effector versus memory cells. Thus, we speculate that this system could also distinguish distinct CD8+ T cell populations.

The pharmacologic inhibition or the genetic deletion of mTOR leads to the generation of Foxp3+ regulatory T cells under otherwise normally activating conditions [14–18]. Consistent with these reports, we observed that under activating conditions, Foxp3+ T cells were enriched within the population of mTOR^lo^ CD4 cells. Furthermore, these data are consistent with previous works demonstrating that low doses of peptide can promote Foxp3+ T regulatory cell generation even in the absence of exogenous TGF-ß [45]. Indeed, we have observed that peptide concentrations that promote T regulatory cell generation fail to induce robust mTOR activation (data not shown). Alternatively, a common technique for generating Foxp3+ regulatory T cells *in vitro* is to robustly stimulate T cells with either high doses of peptide or anti-CD3 in the presence of exogenous TGF-ß [46]. Under such conditions we observe high mTOR activity (**Figs. 7A and S7**). T cells generated in TGF-ß culture conditions have differential metabolic and survival potentials than T cells generated in the presence of rapamycin (**Fig. 7**). Furthermore, Foxp3+ T cells generated from rapamycin versus TGF-ß cultures have distinct phenotypes (**Fig. 7E**). These data lead us to propose that Foxp3+ regulatory T cell fate/ function may be determined by level of mTOR activity. We suggest that Foxp3+ mTOR^hi^ cells resemble short-lived “effector regulatory” T cells characterized by increased glycolysis, increased CTLA-4 and CD39 expression, and decreased Bcl-2. Alternatively, we propose that mTOR^lo^ Foxp3+ cells behave more like long-lived “memory regulatory” cells characterized by decreased glycolytic flux, but enhanced CD25, GITR and Bcl-2 expression. Such a model is consistent with recently published reports describing effector and central regulatory T cell populations [47]. Furthermore, this distinction might help reconcile seemingly conflicting reports in the literature regarding the necessity of mTOR inhibition versus mTOR activation in T regulatory cell [48,49]. Interestingly, the ability to distinguish subsets of regulatory T cells based on size/mTOR activity was recapitulated in human cells CD4+ T cells as well (**Fig. 8**).

Overall, our ability to track the fate of mTOR^hi^ and mTOR^lo^ cells following the activation of naïve T cells reveals a new mTOR/metabolism-centric model of T cell activation [42]. In this model, within a clonal population of T cells, the level of mTOR activation, in part through the upregulation of selective metabolic programs, helps to determine the effector versus memory fate of T cells upon antigen recognition. We believe that this new perspective helps to better define the fate and function of memory CD4+ T cells. Likewise, our model posits the existence of distinct subsets of regulatory T cells.

## Supporting Information

S1 Fig mTORC1 activity correlates with cellular size upon TCR stimulation in a time dependent manner. Splenocytes from a 5c.c7 Rag^–/—^TCR transgenic mouse were stimulated with 0.5ug/ml PCC peptide. A time course assessment of forward scatter (FSC) and mTORC1 activity, indicated by phosphorylated levels of S6 Kinase or ribosomal S6, was measured by flow cytometry. Plots were gated from CD4+ T cells. The data are representative of two independent experiments.
(TIFF)

S2 Fig mTOR^lo^ and mTOR^hi^ CD4+ T cells display no significant difference in cell cycle stage at the time of isolation. a) Splenocytes from 5c.c7 Rag^–/—^mice were stimulated with 0.5ug/ml PCC peptide for 24hrs and then sorted into CD4+CD69+ populations and further separated into FSC/SSC “big” mTOR^hi^ or FSC/SSC “small” mTOR^lo^ populations. Cells were immediately fixed, and DNA content was determined by PI staining. mTOR^hi^ and mTOR^lo^ CD4+ T cells exhibited no significant difference in cell cycle stage at this time post stimulation. b) CFSE labeled 5c.c7 Rag^–/—^splenocytes were stimulated with PCC for 24hrs and activated (CD69+) CD4+ cells were sorted based on 4 size profiles as in Fig. 4C. An illustration of cell size after sort is depicted above the flow plots for clarification of the sorted quartile populations. Top panels depict CFSE vs FSC of each of the 4 sorted populations immediately following the sort. Gates show smallest and largest populations based on Quartiles 1&4 immediately following the sort. Bottom panels depict CFSE vs FSC for each population after culture in IL-2 supplemented media for 3 days. Gate shows percentage of cells with highest CFSE expression. The data are representative of 3 independent experiments.
(TIFF)

S3 Fig Rapamycin treated CD4+T cells exhibit a lower Extracellular Acidification Rate (ECAR) but higher Spare Respiratory Capacity (SRC) than untreated controls. 5c.c7 Rag^–/—^CD4+ T cells stimulated with PCC peptide and treated with 500nM rapamycin exhibit a lower ECAR (a), but higher SRC (b) than untreated controls after 48hrs of stimulation.
(TIFF)

S4 Fig Activated mTOR^lo^ cells have a reduced proliferative capacity compared to mTOR^hi^ cells but can generate Foxp3+ cells in any division. a-b) CFSE labeled 5c.c7 Rag^–/—^splenocytes were stimulated with PCC for 24hrs and activated (CD69+) CD4+ cells were sorted based on 4 size profiles as in Fig. 4C. An illustration of cell size after sort is depicted above flow plots for clarification of sorted quartile populations. After the sort, cells were cultured in IL-2 supplemented media for 3 days, and a) CD25, or b) Foxp3 protein levels were detected by surface or intracellular staining and plotted against CFSE dilution. Gates were determined based on the isotype control staining (left panels). The data are representative of 3 independent experiments.
(TIFF)

S5 Fig Sorted mTOR^lo^ CD4+ T cells from non-TCR transgenic mice preferentially develop into Foxp3+ regulatory cells. a-b) Splenocytes from a C57BL/6 mouse were stimulated with 1ug/ml anti-CD3 for 24 hrs before being sorted into CD4+CD69+ FSC/SSC “big” mTOR^hi^ and “small” mTOR^lo^ populations. Sorted cells were cultured in media supplemented with IL-2 for 4 days, and then analyzed for CD4 and Foxp3 expression by flow cytometry. b) The FACs plots show the percentage of Foxp3+ cells obtained from the sorted mTOR^hi^ and mTOR^lo^ ‘suppressor’ populations used in the suppression assay depicted in Fig. 5E. C) The histograms depict the CTLA-4 expression of the Foxp3+ or Foxp3- populations gated in b. The CTLA-4 MFI is shown in the upper corner of the FACs plot. The data are representative of 3 independent experiments.
(TIFF)

S6 Fig Foxp3+ T cells are de novo generated upon TCR stimulation in conditions of low mTORC1 activation. a-d) Splenocytes from WT Foxp3GFP+ mice were stimulated with 0.1ug/ml anti-CD3 for 20 hrs, and then sorted into CD4+CD69+GFP negative FSC/SSC “big” mTOR^hi^ and “small” mTOR^lo^ populations. a) A schematic of the sorting strategy used. b-d) Sorted cells were cultured in media supplemented with IL-2 for 3 days. b) Foxp3+ expression was determined by flow cytometry. c) FACs plots show the dilution of eFluor670 labeled naive CD4+ ‘responder’ cells after 72hrs of stimulation in co-culture (2:1 responder: suppressor) with mTOR^hi^ or mTOR^lo^ cells. d) The histograms depict the CTLA-4 expression of the Foxp3+ or Foxp3 negative populations gated in b. The CTLA-4 MFI is shown in the upper corner of the FACs plot. The data are representative of at least 3 independent experiments.
(TIFF)

S7 Fig TGF-ß does not inhibit mTOR signaling in activated CD4+ T cells. Splenocytes from a 5c.c7 Rag^–/—^mouse were stimulated with anti-CD3 and anti-CD28 for 4hrs in the presence or absence of 5ng/ml TGF-ß or 500nM rapamycin. Cells were then lysed, and mTOR activity was determined by western blot.
(TIFF)

## References

[1] Brown EJ, Albers MW, Shin TB, Ichikawa K, Keith CT, Lane WS, et al A mammalian protein targeted by G1-arresting rapamycin-receptor complex. Nature. 1994;369(6483):756–8. Epub 1994/06/30. 10.1038/369756a0 . 8008069

[2] Laplante M, Sabatini DM. mTOR signaling at a glance. Journal of cell science. 2009;122(Pt 20):3589–94. Epub 2009/10/09. 10.1242/jcs.051011 19812304 PMC2758797

[3] Laplante M, Sabatini DM. mTOR signaling in growth control and disease. Cell. 2012;149(2):274–93. Epub 2012/04/17. 10.1016/j.cell.2012.03.017 22500797 PMC3331679

[4] Yamagata K, Sanders LK, Kaufmann WE, Yee W, Barnes CA, Nathans D, et al rheb, a growth factor- and synaptic activity-regulated gene, encodes a novel Ras-related protein. The Journal of biological chemistry. 1994;269(23):16333–9. . 8206940

[5] Zhang Y, Gao X, Saucedo LJ, Ru B, Edgar BA, Pan D. Rheb is a direct target of the tuberous sclerosis tumour suppressor proteins. Nature cell biology. 2003;5(6):578–81. Epub 2003/05/29. 10.1038/ncb999 . 12771962

[6] Thoreen CC, Chantranupong L, Keys HR, Wang T, Gray NS, Sabatini DM. A unifying model for mTORC1-mediated regulation of mRNA translation. Nature. 2012;485(7396):109–13. Epub 2012/05/04. 10.1038/nature11083 22552098 PMC3347774

[7] West MJ, Stoneley M, Willis AE. Translational induction of the c-myc oncogene via activation of the FRAP/TOR signalling pathway. Oncogene. 1998;17(6):769–80. Epub 1998/08/26. 10.1038/sj.onc.1201990 . 9715279

[8] Shi Y, Sharma A, Wu H, Lichtenstein A, Gera J. Cyclin D1 and c-myc internal ribosome entry site (IRES)-dependent translation is regulated by AKT activity and enhanced by rapamycin through a p38 MAPK- and ERK-dependent pathway. The Journal of biological chemistry. 2005;280(12):10964–73. Epub 2005/01/07. 10.1074/jbc.M407874200 . 15634685

[9] Hudson CC, Liu M, Chiang GG, Otterness DM, Loomis DC, Kaper F, et al Regulation of hypoxia-inducible factor 1alpha expression and function by the mammalian target of rapamycin. Molecular and cellular biology. 2002;22(20):7004–14. Epub 2002/09/21. 12242281 10.1128/MCB.22.20.7004-7014.2002 PMC139825

[10] Fingar DC, Salama S, Tsou C, Harlow E, Blenis J. Mammalian cell size is controlled by mTOR and its downstream targets S6K1 and 4EBP1/eIF4E. Genes & development. 2002;16(12):1472–87. Epub 2002/06/25. 10.1101/gad.995802 12080086 PMC186342

[11] Waickman AT, Powell JD. Mammalian target of rapamycin integrates diverse inputs to guide the outcome of antigen recognition in T cells. J Immunol. 2012;188(10):4721–9. Epub 2012/05/05. 10.4049/jimmunol.1103143 22556133 PMC3347776

[12] Powell JD, Pollizzi KN, Heikamp EB, Horton MR. Regulation of immune responses by mTOR. Annual review of immunology. 2012;30:39–68. Epub 2011/12/06. 10.1146/annurev-immunol-020711-075024 22136167 PMC3616892

[13] Pollizzi KN, Powell JD. Integrating canonical and metabolic signalling programmes in the regulation of T cell responses. Nature reviews Immunology. 2014;14(7):435–46. Epub 2014/06/26. nri3701 [pii] 10.1038/nri3701 . 24962260 PMC4390057

[14] Delgoffe GM, Kole TP, Zheng Y, Zarek PE, Matthews KL, Xiao B, et al The mTOR kinase differentially regulates effector and regulatory T cell lineage commitment. Immunity. 2009;30(6):832–44. 10.1016/j.immuni.2009.04.014 19538929 PMC2768135

[15] Kang J, Huddleston SJ, Fraser JM, Khoruts A. De novo induction of antigen-specific CD4+CD25+Foxp3+ regulatory T cells in vivo following systemic antigen administration accompanied by blockade of mTOR. Journal of leukocyte biology. 2008;83(5):1230–9. Epub 2008/02/14. 10.1189/jlb.1207851 . 18270248

[16] Haxhinasto S, Mathis D, Benoist C. The AKT-mTOR axis regulates de novo differentiation of CD4+Foxp3+ cells. The Journal of experimental medicine. 2008;205(3):565–74. Epub 2008/02/20. 10.1084/jem.20071477 18283119 PMC2275380

[17] Zeiser R, Leveson-Gower DB, Zambricki EA, Kambham N, Beilhack A, Loh J, et al Differential impact of mammalian target of rapamycin inhibition on CD4+CD25+Foxp3+ regulatory T cells compared with conventional CD4+ T cells. Blood. 2008;111(1):453–62. Epub 2007/10/31. 10.1182/blood-2007-06-094482 17967941 PMC2200823

[18] Sauer S, Bruno L, Hertweck A, Finlay D, Leleu M, Spivakov M, et al. T cell receptor signaling controls Foxp3 expression via PI3K, Akt, and mTOR. Proc Natl Acad Sci U S A. 2008. . 18509048 10.1073/pnas.0800928105 PMC2409380

[19] Delgoffe GM, Pollizzi KN, Waickman AT, Heikamp E, Meyers DJ, Horton MR, et al The kinase mTOR regulates the differentiation of helper T cells through the selective activation of signaling by mTORC1 and mTORC2. Nature immunology. 2011;12(4):295–303. Epub 2011/03/02. 10.1038/ni.2005 21358638 PMC3077821

[20] Heikamp EB, Patel CH, Collins S, Waickman A, Oh MH, Sun IH, et al The AGC kinase SGK1 regulates TH1 and TH2 differentiation downstream of the mTORC2 complex. Nature immunology. 2014;15(5):457–64. Epub 2014/04/08. 10.1038/ni.2867 . 24705297 PMC4267697

[21] Araki K, Turner AP, Shaffer VO, Gangappa S, Keller SA, Bachmann MF, et al mTOR regulates memory CD8 T-cell differentiation. Nature. 2009;460(7251):108–12. Epub 2009/06/23. 10.1038/nature08155 19543266 PMC2710807

[22] He S, Kato K, Jiang J, Wahl DR, Mineishi S, Fisher EM, et al Characterization of the metabolic phenotype of rapamycin-treated CD8 T cells with augmented ability to generate long-lasting memory cells. PLoS One. 2011;6(5):e20107 Epub 2011/05/26. 10.1371/journal.pone.0020107 PONE-D-10-04534 [pii]. 21611151 PMC3096660

[23] Rao RR, Li Q, Odunsi K, Shrikant PA. The mTOR kinase determines effector versus memory CD8+ T cell fate by regulating the expression of transcription factors T-bet and Eomesodermin. Immunity. 2010;32(1):67–78. Epub 2010/01/12. 10.1016/j.immuni.2009.10.010 . 20060330 PMC5836496

[24] Hernandez O, Way S, McKenna J 3rd, Gambello MJ. Generation of a conditional disruption of the Tsc2 gene. Genesis. 2007;45(2):101–6. Epub 2007/01/25. 10.1002/dvg.20271 . 17245776

[25] Delgoffe GM, Kole TP, Cotter RJ, Powell JD. Enhanced interaction between Hsp90 and raptor regulates mTOR signaling upon T cell activation. Molecular immunology. 2009;46(13):2694–8. Epub 2009/07/10. 10.1016/j.molimm.2009.05.185 19586661 PMC2768125

[26] Lee CH, Inoki K, Guan KL. mTOR pathway as a target in tissue hypertrophy. Annual review of pharmacology and toxicology. 2007;47:443–67. Epub 2006/09/14. 10.1146/annurev.pharmtox.47.120505.105359 . 16968213

[27] Feldman ME, Apsel B, Uotila A, Loewith R, Knight ZA, Ruggero D, et al Active-site inhibitors of mTOR target rapamycin-resistant outputs of mTORC1 and mTORC2. PLoS biology. 2009;7(2):e38 Epub 2009/02/13. 10.1371/journal.pbio.1000038 19209957 PMC2637922

[28] Duvel K, Yecies JL, Menon S, Raman P, Lipovsky AI, Souza AL, et al Activation of a metabolic gene regulatory network downstream of mTOR complex 1. Molecular cell. 2010;39(2):171–83. Epub 2010/07/31. 10.1016/j.molcel.2010.06.022 20670887 PMC2946786

[29] van der Windt GJ, Everts B, Chang CH, Curtis JD, Freitas TC, Amiel E, et al Mitochondrial respiratory capacity is a critical regulator of CD8+ T cell memory development. Immunity. 2012;36(1):68–78. Epub 2011/12/31. 10.1016/j.immuni.2011.12.007 22206904 PMC3269311

[30] Choi SW, Gerencser AA, Nicholls DG. Bioenergetic analysis of isolated cerebrocortical nerve terminals on a microgram scale: spare respiratory capacity and stochastic mitochondrial failure. Journal of neurochemistry. 2009;109(4):1179–91. Epub 2009/06/13. 10.1111/j.1471-4159.2009.06055.x 19519782 PMC2696043

[31] Akbar AN, Borthwick NJ, Wickremasinghe RG , Panayoitidis P, Pilling D, Bofill M, et al Interleukin-2 receptor common gamma-chain signaling cytokines regulate activated T cell apoptosis in response to growth factor withdrawal: selective induction of anti-apoptotic (bcl-2, bcl-xL) but not pro-apoptotic (bax, bcl-xS) gene expression. European journal of immunology. 1996;26(2):294–9. Epub 1996/02/01. 10.1002/eji.1830260204 . 8617294

[32] Groom JR, Luster AD. CXCR3 in T cell function. Experimental cell research. 2011;317(5):620–31. Epub 2011/03/08. 10.1016/j.yexcr.2010.12.017 21376175 PMC3065205

[33] Kuczma M, Podolsky R, Garge N, Daniely D, Pacholczyk R, Ignatowicz L, et al Foxp3-deficient regulatory T cells do not revert into conventional effector CD4+ T cells but constitute a unique cell subset. J Immunol. 2009;183(6):3731–41. Epub 2009/08/28. 10.4049/jimmunol.0800601 19710455 PMC2771373

[34] Fu S, Zhang N, Yopp AC, Chen D, Mao M, Chen D, et al TGF-beta induces Foxp3 + T-regulatory cells from CD4 + CD25—precursors. American journal of transplantation: official journal of the American Society of Transplantation and the American Society of Transplant Surgeons. 2004;4(10):1614–27. Epub 2004/09/16. 10.1111/j.1600-6143.2004.00566.x . 15367216

[35] Cheng T, Rodrigues N, Shen H, Yang Y, Dombkowski D, Sykes M, et al Hematopoietic stem cell quiescence maintained by p21cip1/waf1. Science. 2000;287(5459):1804–8. Epub 2000/03/10. . 10710306 10.1126/science.287.5459.1804

[36] Zhang L, Zhang H, Li L, Xiao Y, Rao E, Miao Z, et al TSC1/2 signaling complex is essential for peripheral naive CD8+ T cell survival and homeostasis in mice. PLoS One. 2012;7(2):e30592 Epub 2012/03/01. 10.1371/journal.pone.0030592 22363451 PMC3283604

[37] Johnson SC, Rabinovitch PS, Kaeberlein M. mTOR is a key modulator of ageing and age-related disease. Nature. 2013;493(7432):338–45. Epub 2013/01/18. 10.1038/nature11861 23325216 PMC3687363

[38] Miskov-Zivanov N, Turner MS, Kane LP, Morel PA, Faeder JR. The duration of T cell stimulation is a critical determinant of cell fate and plasticity. Science signaling. 2013;6(300):ra97 Epub 2013/11/07. 10.1126/scisignal.2004217 24194584 PMC4074924

[39] van Panhuys N, Klauschen F, Germain RN. T-cell-receptor-dependent signal intensity dominantly controls CD4(+) T cell polarization In Vivo. Immunity. 2014;41(1):63–74. Epub 2014/07/02. 10.1016/j.immuni.2014.06.003 24981853 PMC4114069

[40] Qiao G, Zhao Y, Li Z, Tang PQ, Langdon WY, Yang T, et al T cell activation threshold regulated by E3 ubiquitin ligase Cbl-b determines fate of inducible regulatory T cells. J Immunol. 2013;191(2):632–9. Epub 2013/06/12. 10.4049/jimmunol.1202068 23749633 PMC3702637

[41] King CG, Koehli S, Hausmann B, Schmaler M, Zehn D, Palmer E. T cell affinity regulates asymmetric division, effector cell differentiation, and tissue pathology. Immunity. 2012;37(4):709–20. Epub 2012/10/23. 10.1016/j.immuni.2012.06.021 23084359 PMC3622938

[42] Powell JD, Heikamp EB, Pollizzi KN, Waickman AT. A Modified Model of T-Cell Differentiation Based on mTOR Activity and Metabolism. Cold Spring Harbor symposia on quantitative biology. 2013. Epub 2013/10/09. 10.1101/sqb.2013.78.020214 24100582 PMC3979500

[43] van der Windt GJ, O'Sullivan D, Everts B, Huang SC, Buck MD, Curtis JD, et al CD8 memory T cells have a bioenergetic advantage that underlies their rapid recall ability. Proc Natl Acad Sci U S A. 2013;110(35):14336–41. Epub 2013/08/14. 10.1073/pnas.1221740110 . 23940348 PMC3761631

[44] Pearce EL, Walsh MC, Cejas PJ, Harms GM, Shen H, Wang LS, et al Enhancing CD8 T-cell memory by modulating fatty acid metabolism. Nature. 2009;460(7251):103–7. Epub 2009/06/06. 10.1038/nature08097 19494812 PMC2803086

[45] Gottschalk RA, Corse E, Allison JP. TCR ligand density and affinity determine peripheral induction of Foxp3 in vivo. The Journal of experimental medicine. 2010;207(8):1701–11. Epub 2010/07/28. 10.1084/jem.20091999 20660617 PMC2916126

[46] Chen W, Jin W, Hardegen N, Lei KJ, Li L, Marinos N, et al Conversion of peripheral CD4+CD25- naive T cells to CD4+CD25+ regulatory T cells by TGF-beta induction of transcription factor Foxp3. The Journal of experimental medicine. 2003;198(12):1875–86. Epub 2003/12/17. 10.1084/jem.20030152 14676299 PMC2194145

[47] Smigiel KS, Richards E, Srivastava S, Thomas KR, Dudda JC, Klonowski KD, et al CCR7 provides localized access to IL-2 and defines homeostatically distinct regulatory T cell subsets. The Journal of experimental medicine. 2014;211(1):121–36. Epub 2014/01/01. 10.1084/jem.20131142 24378538 PMC3892972

[48] Zeng H, Yang K, Cloer C, Neale G, Vogel P, Chi H. mTORC1 couples immune signals and metabolic programming to establish T(reg)-cell function. Nature. 2013;499(7459):485–90. Epub 2013/07/03. 10.1038/nature12297 . 23812589 PMC3759242

[49] Procaccini C, De Rosa V, Galgani M, Abanni L, Cali G, Porcellini A, et al An oscillatory switch in mTOR kinase activity sets regulatory T cell responsiveness. Immunity. 2010;33(6):929–41. Epub 2010/12/15. 10.1016/j.immuni.2010.11.024 21145759 PMC3133602

