# Supplementary figures and images for: Cellular Size as a Means of Tracking mTOR Activity and Cell Fate of CD4+ T Cells upon Antigen Recognition
Source: PLoS One. 2015 Apr 7;10(4):e0121710. doi: 10.1371/journal.pone.0121710 (PMC4388710; doi:10.1371/journal.pone.0121710)

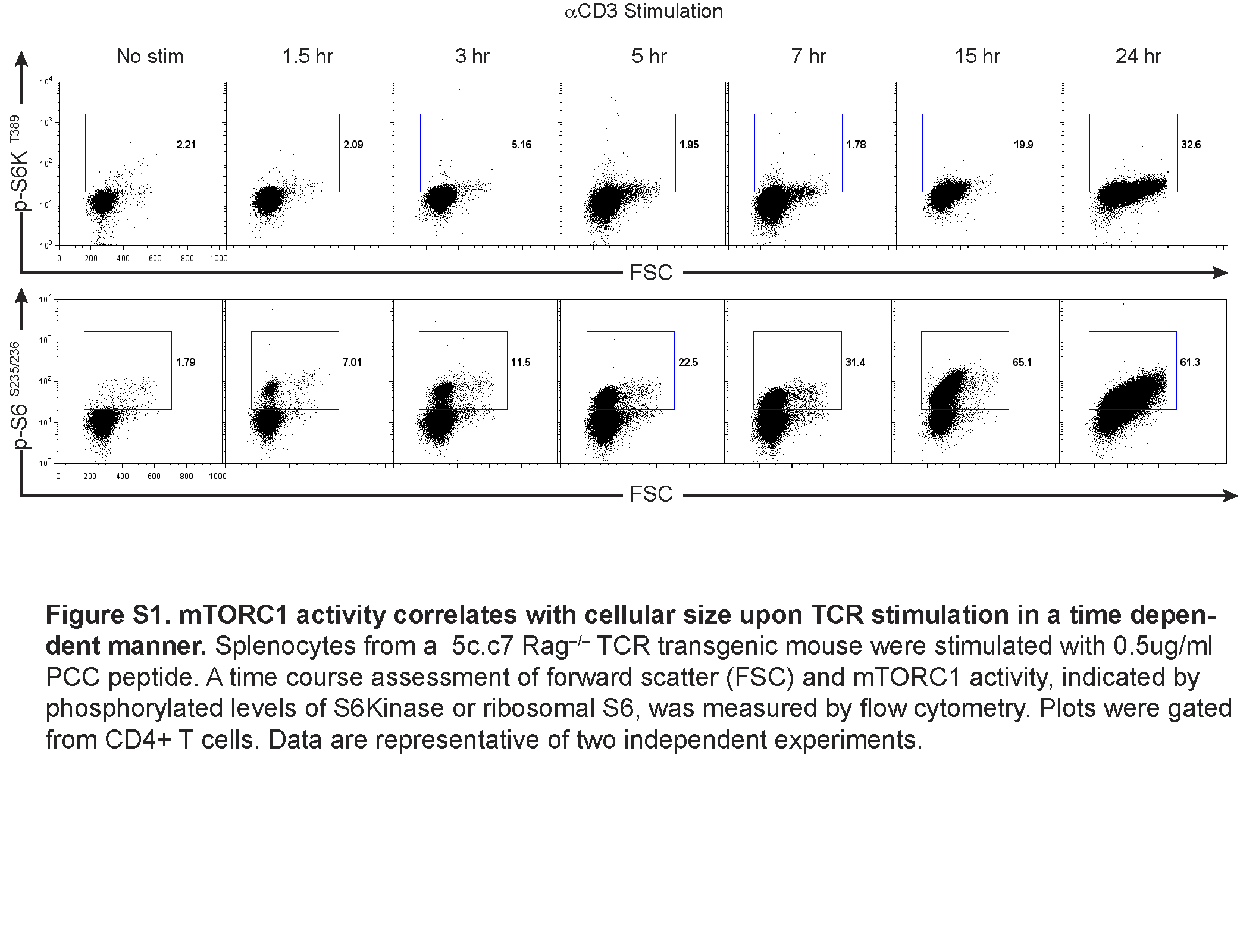

Supplement: S1 Fig — Splenocytes from a 5c.c7 Rag–/—TCR transgenic mouse were stimulated with 0.5ug/ml PCC peptide. A time course assessment of forward scatter (FSC) and mTORC1 activity, indicated by phosphorylated levels of S6 Kinase or ribosomal S6, was measured by flow cytometry. Plots were gated from CD4+ T cells. The data are representative of two independent experiments. (TIFF) [file pone.0121710.s001.tiff]

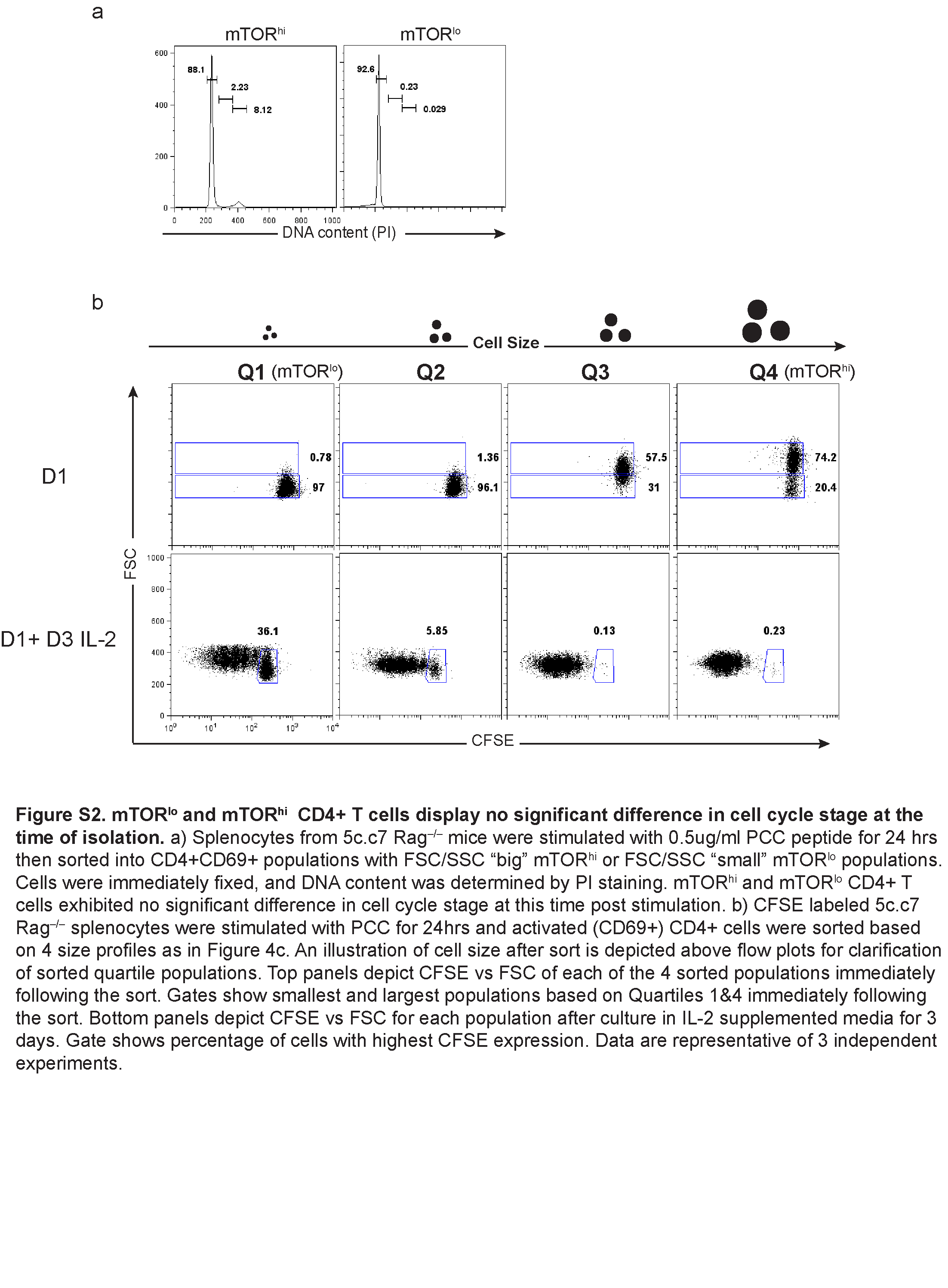

Supplement: S2 Fig — a) Splenocytes from 5c.c7 Rag–/—mice were stimulated with 0.5ug/ml PCC peptide for 24hrs and then sorted into CD4+CD69+ populations and further separated into FSC/SSC “big” mTORhi or FSC/SSC “small” mTORlo populations. Cells were immediately fixed, and DNA content was determined by PI staining. mTORhi and mTORlo CD4+ T cells exhibited no significant difference in cell cycle stage at this time post stimulation. b) CFSE labeled 5c.c7 Rag–/—splenocytes were stimulated with PCC for 24hrs and activated (CD69+) CD4+ cells were sorted based on 4 size profiles as in Fig. 4C. An illustration of cell size after sort is depicted above the flow plots for clarification of the sorted quartile populations. Top panels depict CFSE vs FSC of each of the 4 sorted populations immediately following the sort. Gates show smallest and largest populations based on Quartiles 1&4 immediately following the sort. Bottom panels depict CFSE vs FSC for each population after culture in IL-2 supplemented media for 3 days. Gate shows percentage of cells with highest CFSE expression. The data are representative of 3 independent experiments. (TIFF) [file pone.0121710.s002.tiff]

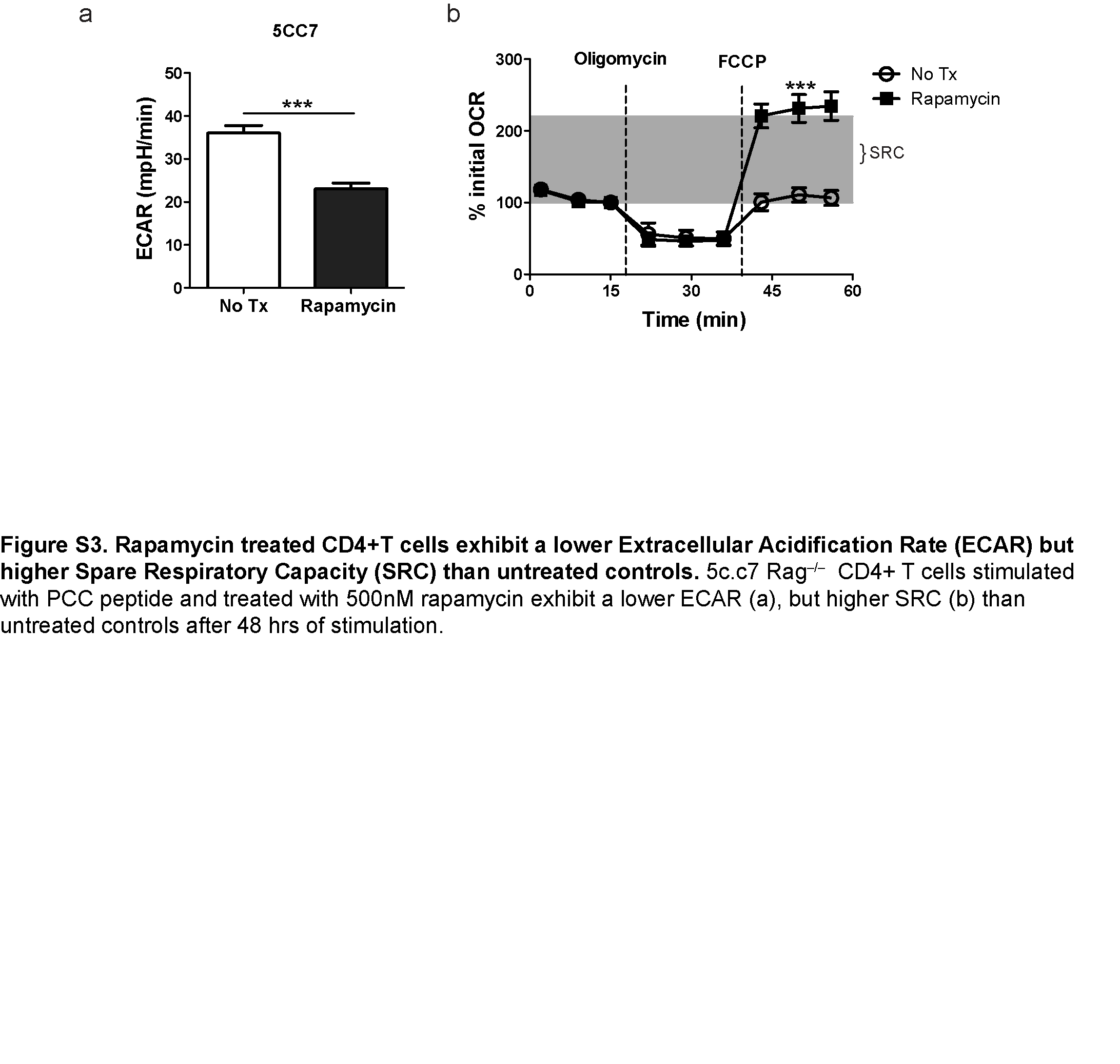

Supplement: S3 Fig — 5c.c7 Rag–/—CD4+ T cells stimulated with PCC peptide and treated with 500nM rapamycin exhibit a lower ECAR (a), but higher SRC (b) than untreated controls after 48hrs of stimulation. (TIFF) [file pone.0121710.s003.tiff]

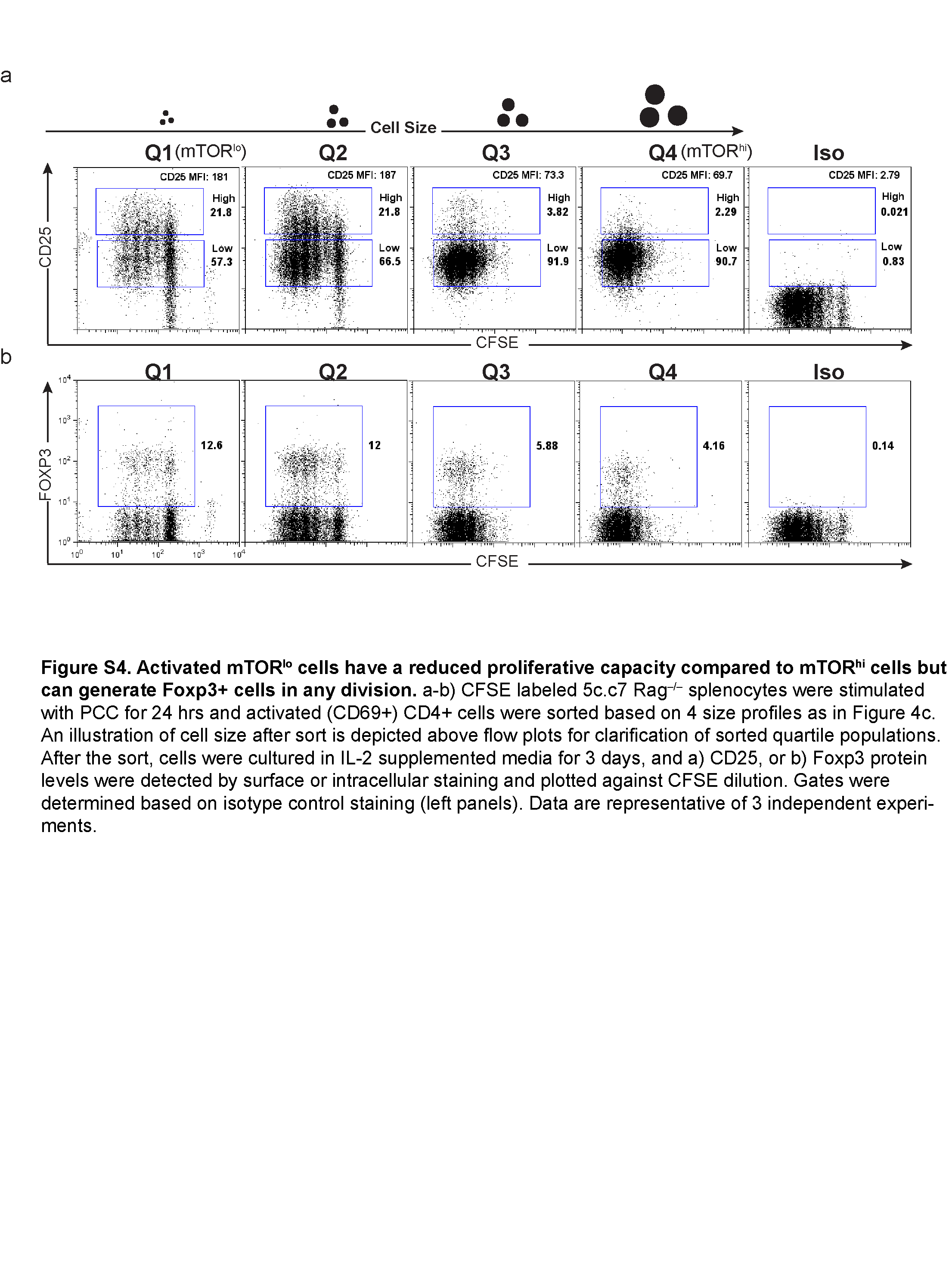

Supplement: S4 Fig — a-b) CFSE labeled 5c.c7 Rag–/—splenocytes were stimulated with PCC for 24hrs and activated (CD69+) CD4+ cells were sorted based on 4 size profiles as in Fig. 4C. An illustration of cell size after sort is depicted above flow plots for clarification of sorted quartile populations. After the sort, cells were cultured in IL-2 supplemented media for 3 days, and a) CD25, or b) Foxp3 protein levels were detected by surface or intracellular staining and plotted against CFSE dilution. Gates were determined based on the isotype control staining (left panels). The data are representative of 3 independent experiments. (TIFF) [file pone.0121710.s004.tiff]

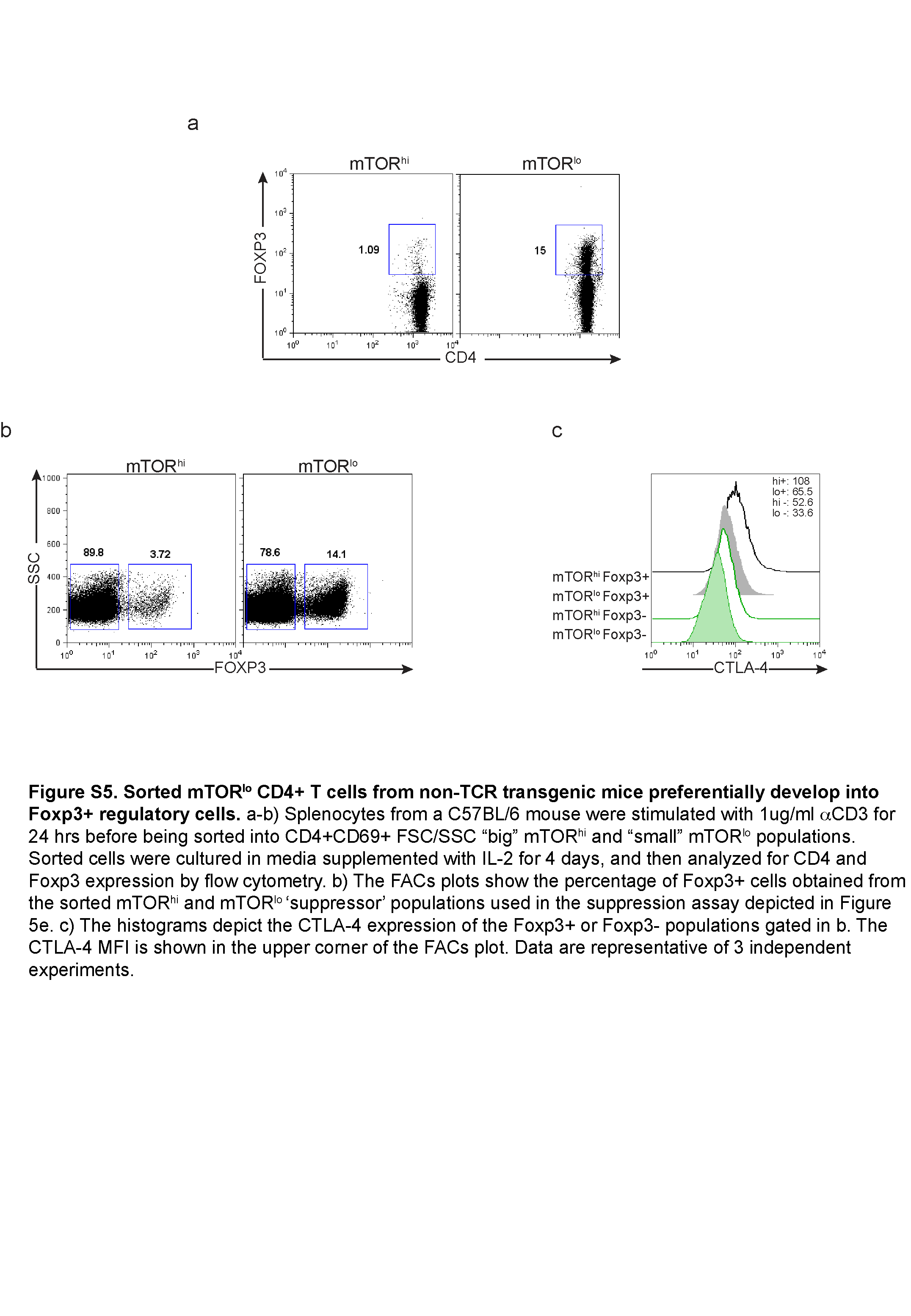

Supplement: S5 Fig — a-b) Splenocytes from a C57BL/6 mouse were stimulated with 1ug/ml anti-CD3 for 24 hrs before being sorted into CD4+CD69+ FSC/SSC “big” mTORhi and “small” mTORlo populations. Sorted cells were cultured in media supplemented with IL-2 for 4 days, and then analyzed for CD4 and Foxp3 expression by flow cytometry. b) The FACs plots show the percentage of Foxp3+ cells obtained from the sorted mTORhi and mTORlo ‘suppressor’ populations used in the suppression assay depicted in Fig. 5E. C) The histograms depict the CTLA-4 expression of the Foxp3+ or Foxp3- populations gated in b. The CTLA-4 MFI is shown in the upper corner of the FACs plot. The data are representative of 3 independent experiments. (TIFF) [file pone.0121710.s005.tiff]

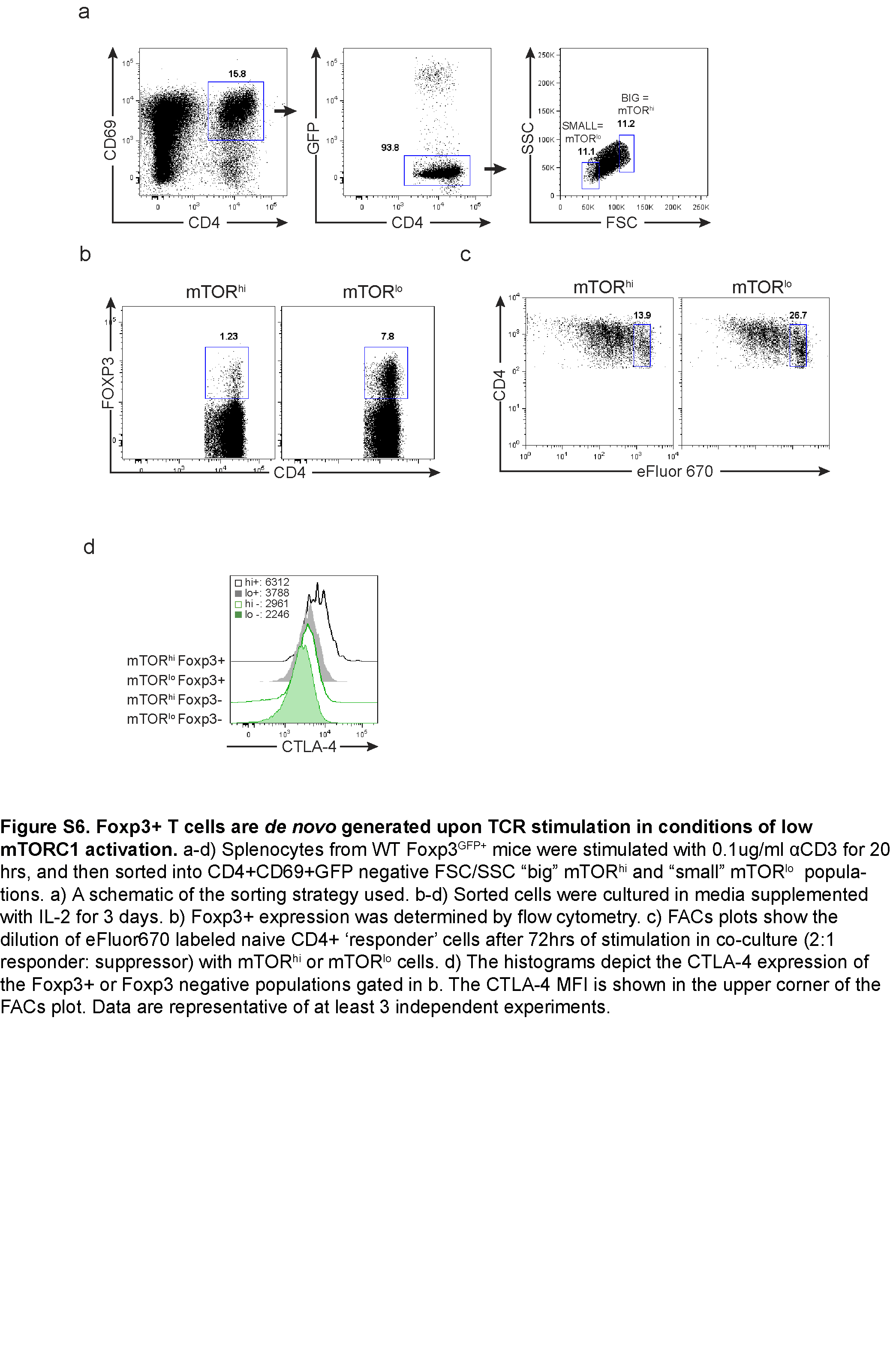

Supplement: S6 Fig — a-d) Splenocytes from WT Foxp3GFP+ mice were stimulated with 0.1ug/ml anti-CD3 for 20 hrs, and then sorted into CD4+CD69+GFP negative FSC/SSC “big” mTORhi and “small” mTORlo populations. a) A schematic of the sorting strategy used. b-d) Sorted cells were cultured in media supplemented with IL-2 for 3 days. b) Foxp3+ expression was determined by flow cytometry. c) FACs plots show the dilution of eFluor670 labeled naive CD4+ ‘responder’ cells after 72hrs of stimulation in co-culture (2:1 responder: suppressor) with mTORhi or mTORlo cells. d) The histograms depict the CTLA-4 expression of the Foxp3+ or Foxp3 negative populations gated in b. The CTLA-4 MFI is shown in the upper corner of the FACs plot. The data are representative of at least 3 independent experiments. (TIFF) [file pone.0121710.s006.tiff]

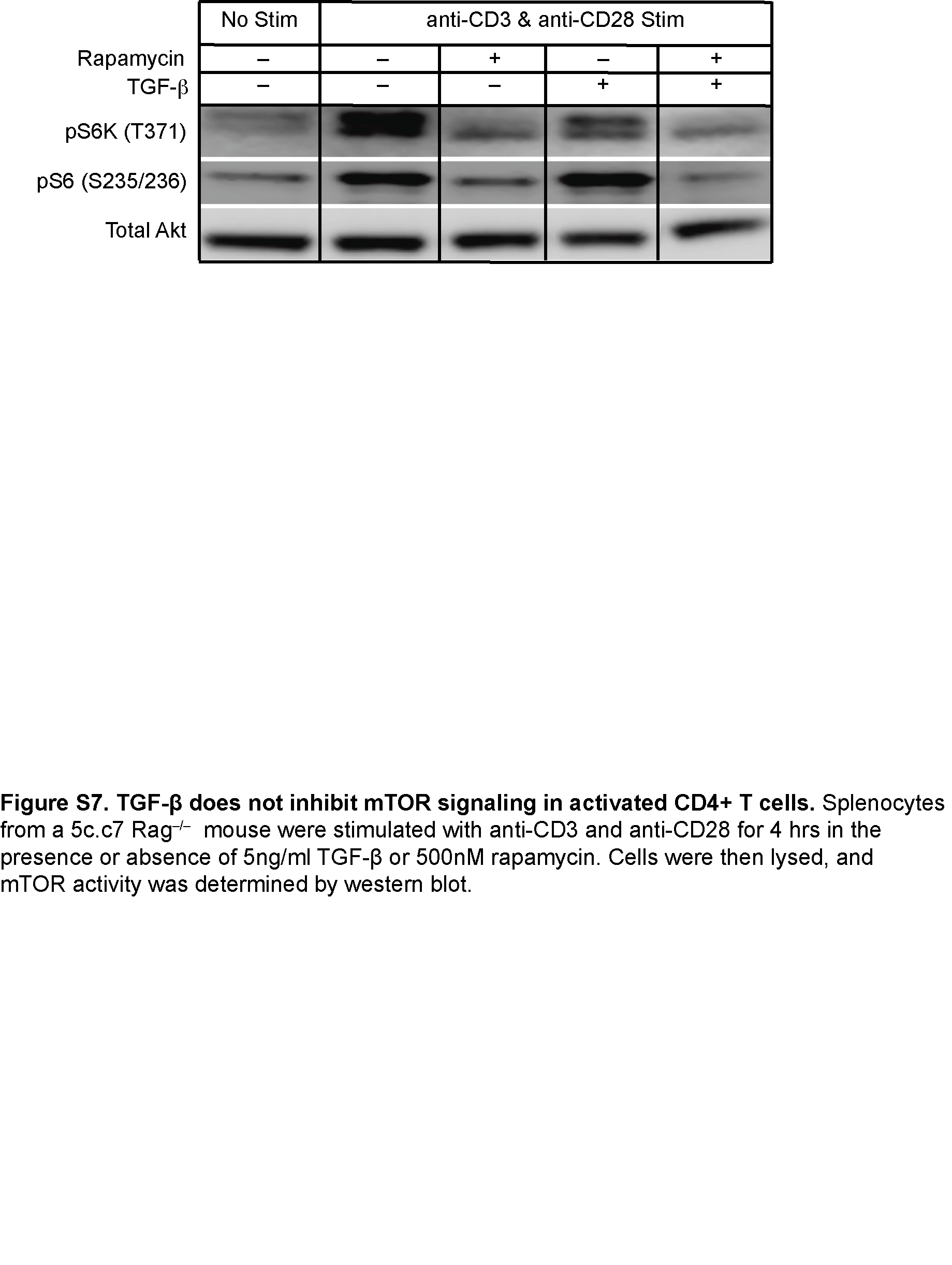

Supplement: S7 Fig — Splenocytes from a 5c.c7 Rag–/—mouse were stimulated with anti-CD3 and anti-CD28 for 4hrs in the presence or absence of 5ng/ml TGF-ß or 500nM rapamycin. Cells were then lysed, and mTOR activity was determined by western blot. (TIFF) [file pone.0121710.s007.tiff]
